# Potassium nutrient status drives posttranslational regulation of a low-K response network in *Arabidopsis*

**DOI:** 10.1038/s41467-023-35906-5

**Published:** 2023-01-23

**Authors:** Kun-Lun Li, Ren-Jie Tang, Chao Wang, Sheng Luan

**Affiliations:** Department of Plant and Microbial Biology, University of California, Berkeley, CA 94720 USA

**Keywords:** Plant signalling, Abiotic, Plant cell biology

## Abstract

Under low-potassium (K^+^) stress, a Ca^2+^ signaling network consisting of calcineurin B-like proteins (CBLs) and CBL-interacting kinases (CIPKs) play essential roles. Specifically, the plasma membrane CBL1/9-CIPK pathway and the tonoplast CBL2/3-CIPK pathway promotes K^+^ uptake and remobilization, respectively, by activating a series of K^+^ channels. While the dual CBL-CIPK pathways enable plants to cope with low-K^+^ stress, little is known about the early events that link external K^+^ levels to the CBL-CIPK proteins. Here we show that K^+^ status regulates the protein abundance and phosphorylation of the CBL-CIPK-channel modules. Further analysis revealed low K^+^-induced activation of VM-CBL2/3 happened earlier and was required for full activation of PM-CBL1/9 pathway. Moreover, we identified CIPK9/23 kinases to be responsible for phosphorylation of CBL1/9/2/3 in plant response to low-K^+^ stress and the HAB1/ABI1/ABI2/PP2CA phosphatases to be responsible for CBL2/3-CIPK9 dephosphorylation upon K^+^-repletion. Further genetic analysis showed that HAB1/ABI1/ABI2/PP2CA phosphatases are negative regulators for plant growth under low-K^+^, countering the CBL-CIPK network in plant response and adaptation to low-K^+^ stress.

## Abstract (web summary)

Potassium is essential for plant growth and development. Here the authors present evidence that plants respond to low potassium availability by modulating the abundance and phosphorylation status of proteins in CBL-CIPK-channel modules.

## Introduction

As an essential macronutrient for plant growth and development, potassium (K^+^) nutrient status in soils has direct consequences on crop yield and quality^1,2^. Since soluble K^+^ in most arable fields is low, crop production relies on extensive use of K^+^ fertilizers^3,4^. However, heavy use of fertilizers is not sustainable because fertilizers are costly and cause environmental pollution^5^. To support sustainable agriculture, a key strategy is to breed crops with improved K^+^ use efficiency so that they can produce more with less input of fertilizers, which requires a thorough understanding of molecular mechanisms that allow plants to respond and adapt to limited K^+^ availability in the soil.

While cellular K^+^ concentration in plants is maintained at approximately 100 mM, the typical K^+^ concentration in natural soils fluctuates between 0.1 and 1 mM^6–10^. After the uptake of K^+^ from the soil into the root symplast and subsequent release into the xylem apoplast, K^+^ is translocated from root to shoot and distributed throughout the plant^11,12^. Once arrived in sink cells, K^+^ is utilized for cellular metabolism and osmoregulation with excess K^+^ sequestered into the vacuole^13,14^. K^+^ efflux from plant cells is also involved in the adjustment of intracellular K^+^ homeostasis in response to altered environment conditions^15^. To deal with K deficiency, plants have evolved two major mechanisms to maintain K^+^ homeostasis in plant cells, one is K^+^ acquisition from the soil and another is K^+^ remobilization from vacuolar stores^7,16–18^. In *Arabidopsis* roots, the high-affinity K^+^ transporter HAK5 (high-affinity K^+^/H^+^ symporter) and the inward-rectifier K^+^ channel AKT1 (Arabidopsis K^+^ transport system 1) are two major contributors to K^+^ uptake under K-limiting conditions in the natural soil^19–25^. The vacuolar K^+^-pool is stocked up in times of sufficiency, but under K^+^ limited environments, several tonoplast K^+^-permeable channels, including two-pore K^+^ (TPK) channels, facilitate K^+^ remobilization from the vacuole to support a stable concentration in the cytoplasm^26^. An interesting question arises: how does a plant cell balance the demand and supply of K^+^ by connecting K^+^ status (as a demand signal) with the activity of channels and transporters (as a supply response)? This question has been addressed by studies on a calcium (Ca^2+^)-dependent signaling mechanism consisting of calcineurin B-like (CBL) calcium sensors and CBL-interacting kinases (CIPKs). A typical CBL-CIPK signaling module is activated by elevation in free cytosolic Ca^2+^ that binds to CBLs through their EF hand motifs and trigger conformational changes. Activated CBLs interact with CIPKs that trans-phosphorylate and modulate the activity of downstream targets such as ion transporters^16,27–34^. In the case of low-K^+^ response, K^+^ deficiency has been shown to triggers distinct Ca^2+^ changes in *Arabidopsis* roots^35^, which may activate CBL-CIPK signaling pathways that in turn promote K^+^-uptake and/or vacuolar remobilization. With respect to K^+^ uptake, two CBL proteins, CBL1 and CBL9, function together with CIPK1/9/23 at the plasma membrane (PM) where they activate K^+^ channels and carriers such as AKT1 and HAK5 through phosphorylation^27–29,36,37^. In parallel, CBL2 and CBL3 recruit four CIPKs, CIPK3/9/23/26, to the vacuolar membrane (VM) where the CBL-CIPK modules initiate K^+^ remobilization by activating transporters including two-pore K^+^-channels (TPKs)^16^. These findings demonstrate that the PM-CBL1/9-CIPK23 and VM-CBL2/3-CIPK3/9/23/26 signaling modules serve as response mechanisms that link the low-K^+^ stress to the activation of the transport activities to maintain K^+^ homeostasis.

Although the dual CBL-CIPK pathways regulating K^+^ channels and transporters have been well established, it remains unknown how these CBL-CIPK proteins are modified in response to changes in K^+^ status in the environment. In this study, we monitored the behaviors of CBL1/9, CBL2/3, CIPK9/23, AKT1, and TPK1 proteins and revealed K^+^ dose- and time-dependent posttranslational modifications of these proteins in response to changing K^+^ nutrient status, establishing previously unanticipated mechanisms for nutrient sensing in plants. The abundance and phosphorylation of these proteins reflect the activity of the dual CBL-CIPK pathways and coincide with the nutrient availability: when K^+^ levels are low, CBL-CIPK-K^+^ channel becomes more abundant and more active to boost K^+^ uptake from soil and vacuole release into the cytosol; when K^+^ levels are high, CBL-CIPK-K^+^ channel activity is not required and thus dephosphorylated and degraded. Interestingly, although CBL1/9 and CBL2/3 are localized to distinct subcellular compartments, CBL2/3 activates prior to and contributes to the accumulation of CBL1/9 proteins, revealing a unique mechanism for crosstalk between PM and VM pathways. Together with our previously described genetic analysis^16^, these biochemical data further support the conclusion that VM-CBL2/3-CIPK pathway functions as a primary mechanism for plants to respond and adapt to low-K^+^ stress.

## Results

### The plasma membrane and vacuolar “CBL-CIPK-channel” modules respond to external K^+^ by altering protein abundance and phosphorylation status

**Fig. 1:**
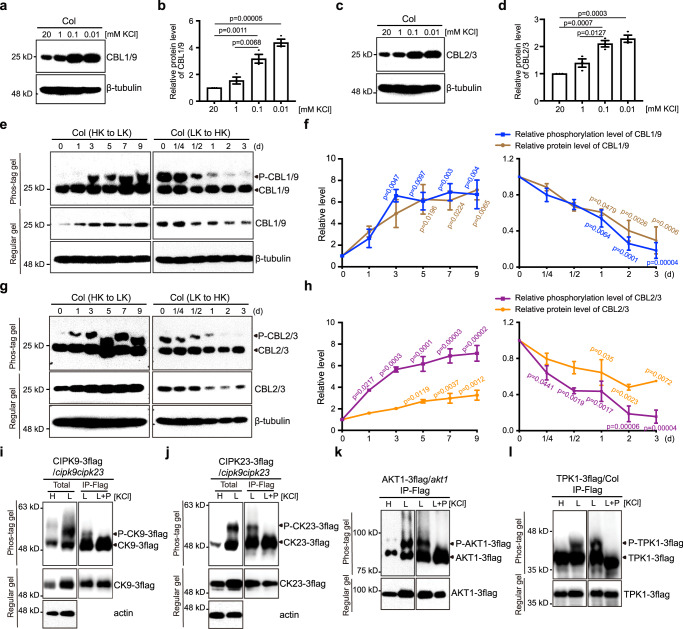
K^+^ status controls the abundance and phosphorylation of CBL1/9-CIPK9/23 and CBL2/3-CIPK9/23 modules. **a**–**d** CBL1/9 protein abundance (**a**, **b**) and CBL2/3 protein abundance (**c**, **d**) in Col plants grown under different external K^+^ concentrations (20, 1, 0.1, 0.01 mM) for 7 days. **e**–**h** The abundance and phosphorylation of CBL1/9 (**e, f**) and CBL2/3 (**g, h**) in Col plants upon high- to low-K^+^ transfer or vice versa. In the left panel, Col seedlings were first grown under high-K^+^ (20 mM) condition for 4 days and then transferred to low-K^+^ (10 μM) medium for the indicated time (d, days). In the right panel, Col seedlings were first grown under low-K^+^ (10 μM) condition for 7 days and then transferred to high-K^+^ (20 mM) medium for the indicated time (d, days). In **b**, **d**, **f**, and **h**, protein abundance of CBLs was normalized against β-tubulin loading control on Regular gel. In **f** and **h**, phosphorylated CBLs were normalized against total CBLs on Phos-tag gel. The value of the starting point (0 d) was set to 1. Quantitative data are shown as means ± s.e.m., *n* = 3 (biologically independent experiments). P values represent statistically significant differences between plants before and after treatment, as calculated by one-way analysis of variance (ANOVA) followed by a Tukey’s multiple comparison test. **i**–**l** The protein level and phosphorylation status of CIPK9-3flag (**i**), CIPK23-3flag (**j**), AKT1-3flag (**k**), TPK1-3flag (**l**) under high-K^+^ or low-K^+^ conditions. Total protein was extracted from seedlings grown under high-K^+^ (20 mM) or low-K^+^ (10 μM) for 7 days. IP-Flag samples were prepared by immunoprecipitation using anti-flag beads and were then treated with or without phosphatase before regular PAGE and phostag-PAGE. actin was used as a loading control. Each immunoblot result is the representative of at least three repeats.

When facing K^+^ deficiency, plants activate the CBL1/9-CIPK23 pathway to boost K^+^-uptake capacity by activating K^+^ channels and transporters in roots^21,27–29^. To address the molecular mechanisms underlying CBL-CIPK activation in response to low-K^+^, we examined the behavior of the CBL proteins to explore the possible processes that couple the external K^+^ status to the function of the calcium sensors. Using a polyclonal antibody against a recombinant CBL1 protein, we monitored the content of native CBL1 and CBL9 proteins in *Arabidopsis* seedlings. Due to high homology and functional redundancy between CBL1 and CBL9, the CBL1 polyclonal antibody reacted with both CBL1 and CBL9 proteins (Supplementary Fig. 1a), as demonstrated by the presence of a protein band at the correct molecular weight in the wild type, *cbl1* or *cbl9* single mutant, but not in the *cbl1/9* double mutant. To determine whether CBL1/9 protein level is regulated in response to the change of external K^+^ levels, we performed western blot analysis using total protein extracted from *Arabidopsis* seedlings grown on the modified MS medium with different K^+^ levels (Fig. 1a, b, Supplementary Fig. 1b, c). Our results suggested that, with a decreasing level of external K^+^ (20, 1, 0.1, 0.01 mM), the primary roots of wild type seedlings shortened, and CBL1/9 protein abundance increased (Fig. 1a, b, supplementary Fig. 1b, c). In particular, a large boost of CBL1/9 protein (about 2.1-fold) occurred from 1 mM to 0.1 mM, physiological K^+^ levels associated with natural soils^9,38–41^. To further determine the kinetic changes of CBL1/9 levels in response to external K^+^ levels, we transferred seedlings from high- to low-K^+^ condition, or vice versa. Interestingly, while the increase in CBL1/9 abundance by high- to low-K^+^ transfer was not detectable until 5 days upon K^+^ deprivation, the decrease of CBL1/9 proteins happened much faster (on the first day after low- to high-K^+^ transfer) and reached the lowest level on the third day (Supplementary Fig. 1d). We reasoned that during the pre-culture under high K^+^ condition, seedlings accumulated excess K^+^, which supplied plants with sufficient K^+^ in the first 7 days upon low-K^+^ treatment, leading to a delayed K^+^-starvation response. In contrast, low- to high-K^+^ transfer immediately allowed starved seedlings to acquire sufficient K^+^, making the CBL1/9 signaling proteins obsolete, and resulting in a more rapid degradation response. These results revealed that the abundance of CBL1/9 proteins is fine-tuned by extracellular K^+^ levels. We also measured CBL1 and CBL9 mRNA levels under high- and low-K^+^ and found no significant differences (Supplementary Fig. 1e), indicating that a posttranslational modification is responsible for the fluctuation in CBL1/9 protein abundance.

In addition to PM-CBL1/9-CIPK23 pathway, VM-CBL2/3-CIPK3/9/23/26 represents another indispensable pathway for plants to cope with K^+^ deficiency^16^. Unlike PM-CBL1/9-CIPK modules which are mainly responsible for boosting K^+^ uptake, VM-CBL2/3-CIPK pathway mediates K^+^ remobilization from vacuoles into cytoplasm by activating tonoplast TPKs channels^16^. Despite the different subcellular locations and functional processes they participate, the PM- and VM-CBL-CIPK pathways are both activated upon K^+^-deficiency. We thus investigated whether the vacuolar CBL2/3 protein levels were, like PM-CBLs, governed by external K^+^ levels as well. CBL2 and CBL3 showed high homology and CBL3 antibody we generated recognized both CBL2 and CBL3 in *Arabidopsis*^42^ (Supplementary Fig. 2a). We examined CBL2/3 protein levels in seedlings grown under various concentrations of K^+^ and found that CBL2/3 protein abundance was enriched without altering transcription level under low-K^+^ stress (Fig. 1c, d, Supplementary Fig. 2b).

Previous work showed that CBL1 and CBL9 proteins are phosphorylated in vitro^43,44^. To monitor if this is the case in plants as a response to K^+^ shortage, we grew wild-type seedlings on sufficient-K^+^ (20 mM K^+^) medium for 4 days and then transferred them to K^+^-deficient (10 μM K^+^) conditions and took samples at five different time points (1, 3, 5, 7 and 9 d). We confirmed that this low-K^+^ treatment was effective by examining the expression level of HAK5, a marker gene in *Arabidopsis* response to K^+^ deficiency^45,46^. As shown in Supplementary Fig. 3, HAK5 transcript level began to increase after 1d of low-K^+^ transfer and showed a striking increase (307-fold) on day 3 and peaked on day 7 (669-fold) (Supplementary Fig. 3). Using Phos-tag mobility shift assay, we found that the level of CBL1/9 phosphorylation was strongly induced on the 3rd day after low-K^+^ transfer, followed by a further increase on day 7 and remained high on day 9 (Fig. 1e, f, left panel). The pattern observed on the up-regulation of HAK5 expression and CBL1/9 phosphorylation suggested that low-K responsive events consistently happened at both the transcriptional level and posttranslational level. Furthermore, low- to high-K^+^ transfer triggered the dephosphorylation of CBL1/9 (Fig. 1e, f, right panel), suggesting that external K^+^ level modulates the phosphorylation status of CBL1/9. Phosphatase treatment confirmed that the mobility shift of CBL1/9 proteins under low-K^+^ treatment resulted from phosphorylation (Supplementary Fig. 4a). We also generated transgenic plants harboring UBQ10: CBL1-3flag or UBQ10: CBL9-3flag construct in the *cbl1/9* double mutant background. In both cases, protein and phosphorylation levels of CBL1-3flag and CBL9-3flag were dramatically elevated by low-K^+^ treatment (Supplementary Fig. 1f, g).

We further examined the phosphorylation status of CBL2/3 under low-K^+^ treatment using the phos-tag gel assay and found that, like PM-CBL1/9, the vacuolar CBL2/3 were also highly phosphorylated under low- K^+^ treatment (Supplementary Fig. 4b). Furthermore, high- to low-K^+^ transfer enhanced the phosphorylation whereas low- to high-K^+^ transfer triggered the dephosphorylation of CBL2/3 (Fig. 1g, h), suggesting that external K^+^ level controls CBL2/3 phosphorylation status. In parallel with the change of phosphorylation, CBL2/3 protein level was also elevated during high- to low-K^+^ transfer and decreased during low- to high-K^+^ transfer (Fig. 1g, h).

As the tonoplast CBL2/3-CIPK3/9/23/26 module also functions in Mg^2+^ homeostasis under high-Mg^2+^ stress^42^, we next examined whether CBL2/3 are phosphorylated in response to high Mg^2+^. Interestingly, high-Mg^2+^ stress (30 mM MgCl_2_) failed to enhance CBL2/3 phosphorylation (Supplementary Fig. 5a). As a further confirmation, we transferred the seedlings from low-K^+^ to high-K^+^ or high-K^+^ plus high-Mg^2+^. If high-Mg^2+^ stress also enhances CBL2/3 phosphorylation as low-K^+^ does, we would expect to see the inhibitory effect of high-Mg^2+^ on high-K^+^-triggered CBL2/3 dephosphorylation. However, this was not the case as the degree of CBL2/3 dephosphorylation was similar between transfer to high-K^+^ or high-K^+^ plus high-Mg^2+^ (Supplementary Fig. 5b). These results indicated that, although CBL2/3 function in both low-K^+^ and high-Mg^2+^ responses, their phosphorylation is regulated in a low-K^+^ stress-specific manner.

CBLs and CIPKs form CBL-CIPK complexes to regulate their downstream target proteins^16,27–29,47,48^. We found that CBL1/9 and CBL2/3 respond to low-K^+^ stress by increasing protein abundance (Fig. 1). For increased levels of CBLs to have functional relevance, we speculate that their partner kinases, CIPK9 and CIPK23, may also respond to K^+^ deficiency in the similar manner. Under low-K^+^ treatment, *CIPK9* mRNA level was elevated dramatically in the wild-type plants (Supplementary Fig. 8a), which is consistent with the previous study^16^. The *CIPK23* mRNA level did not change significantly under low-K^+^ stress condition used in this study (Supplementary Fig. 8b), which differs from the previous findings that *CIPK23* mRNA level is induced by low-K^+^ treatment^27,36,37^. Such discrepancy may result from variations in experimental conditions and developmental stages of plant materials. To exclude the contribution of transcriptional control, we generated transgenic plants harboring UBQ10: CIPK9-3flag or UBQ10: CIPK23-3flag construct in the *cipk9/23* double mutant background. In both cases, CIPK9-3flag and CIPK23-3flag were functionally equivalent to the native proteins because they rescued the low-K^+^-sensitive phenotype in *cipk9/23* double mutant (Supplementary Figs. 6, 7). In western blot analyses using flag-antibody, we found that protein levels of CIPK9-3flag and CIPK23-3flag were dramatically elevated by low-K^+^ treatment (Supplementary Fig. 8c, d). Moreover, both CIPK9-3flag and CIPK23-3flag proteins in seedlings grown under low-K^+^ treatment became up-shifted in the phos-tag gel, and the mobility shift was removed by phosphatase treatment (Fig. 1i, j), confirming that low-K^+^ stress enhances the phosphorylation of CIPK9 and CIPK23 in plants.

Previous studies demonstrated that CIPK9/23, like many other kinases, can auto-phosphorylate in vitro^27–29,49^. We examined whether low-K^+^-induced CIPK9/23 phosphorylation was auto-catalyzed *in planta*. To this end, we introduced the kinase-dead version of CIPK9 (CIPK9^K48N^)^16,49^ or CIPK23 (CIPK23^K60N^)^28^ fused with 3x flag tag into *cipk9/23* double mutant. To ensure correct CIPK9^K48N^−3flag and CIPK23^K60N^−3flag transgenes were introduced, we sequenced the genomic DNA from several transgenic lines and confirmed that all UBQ10: CIPK9^K48N^−3flag plants contained the K48N mutation (Supplementary Fig. 9a) and all UBQ10: CIPK23^K60N^−3flag plants contained the K60N mutation (Supplementary Fig. 9b). To test if the kinase-dead versions of CIPKs were indeed inactive in vivo, we analyzed CBL1/9 phosphorylation in the transgenic lines expressing similar level of wild type CIPK or kinase-dead CIPK. Our results showed that low-K^+^-induced CBL1/9 phosphorylation was dramatically reduced in transgenic seedlings expressing the kinase-dead version of CIPK 9 (UBQ10: CIPK9^K48N^−3flag) as compared with UBQ10: CIPK9-3flag control (Supplementary Fig. 9c). Similar result was observed when comparing the seedlings expressing wild type CIPK23-3flag versus kinase-dead CIPK23^K60N^−3flag (Supplementary Fig. 9d). These results suggested that the kinase activity was ablated in the CIPK9^K48N^ or CIPK23^K60N^ mutants in plants. We next investigated whether low-K^+^-induced phosphorylation of CIPK9/23 results from auto-activity of the kinases by comparing seedlings expressing wild type or kinase-dead version of CIPK9 or CIPK23. Interestingly, the phosphorylation levels of CIPK9^K48N^−3flag or CIPK23^K60N^−3flag, despite their lack of kinase activity, were phosphorylated at a similar level as their wild type controls in response to low-K^+^ treatment (Supplementary Fig. 9e, f). These results suggested that low-K^+^-induced phosphorylation of CIPK9 and CIPK23 was catalyzed by other kinase(s) in plants.

Although previous studies show that PM-CBL-CIPK pathway phosphorylates and activates AKT1 channel in *Xenopus* oocyte^27,28^, it remains unknown if AKT1 protein is phosphorylated *in planta* as a response to low-K^+^ stress. Further, our finding of low-K^+^-induced increase in protein abundance of both CBLs and CIPKs prompted us to examine the abundance of their target channels such as AKT1. As done with CBLs and CIPKs, we constructed UBQ10: AKT1-3flag construct and introduced it into *akt1* mutant. The tagged version of AKT1 was functional as shown by its capability in complementing *akt1* mutant phenotype under low-K^+^ stress (Supplementary Fig. 10). As shown by western blot, AKT1-3flag protein accumulated at a much higher level when seedlings were grown under low-K^+^ conditions (Supplementary Fig. 11a). Using phos-tag assay, we found that AKT1-3flag proteins also became up-shifted in the gel when plants were grown under low-K^+^ conditions and the mobility shift was removed by phosphatase (Fig. 1k), confirming that low-K^+^ stress enhances the *in planta* phosphorylation of AKT1.

As TPKs serve as the target channels of VM-CBL2/3-CIPKs module to activate K^+^ efflux from vacuole^16^, we examined whether TPKs channels were, like AKT1, also phosphorylated by low-K^+^ stress using TPK1 as an example. We introduced 3x flag epitope-tagged TPK1 into Col background and found that K^+^ starvation clearly enhanced TPK1 phosphorylation status (Fig. 1l). Interestingly, abundance of TPK1 was not affected by low-K^+^ treatment (Supplementary Fig. 11b), suggesting that the abundance of TPK1 may not be limiting and phosphorylation holds the key for activation of the channel.

### CIPK9/23 are required for low-K-induced phosphorylation of both PM- and VM-localized CBLs

**Fig. 2:**
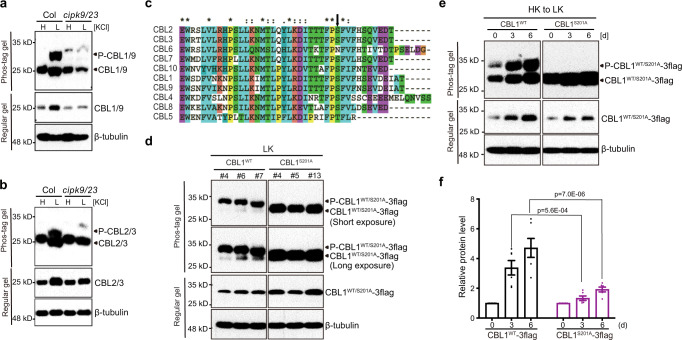
CIPK9/23 are responsible for enhanced phosphorylation levels of CBL1/9/2/3 proteins under low-K^+^ stress and CBL1/9 phosphorylation contributes to protein accumulation under low-K^+^ stress. **a**, **b** Phosphorylation levels of CBL1/9 (**a**) and CBL2/3 (**b**) in Col and *cipk9/23* double mutant grown under high-K^+^ (20 mM) or low-K^+^ (10 μM) for 7 days. **c** Amino acid sequence alignment of the C-terminus among ten *Arabidopsis* CBL members. The alignment is generated with Clustal X software. S201 site is indicated by a black arrow. **d** Phosphorylation status of CBL1^WT^−3flag or CBL1^S201A^−3flag under low-K^+^ stress. Transgenic plants expressing flag-tagged CBL1 wild type (CBL1^WT^) or site-directed mutant CBL1^S201A^ in the *cbl1cbl9* background were grown under low-K^+^ (10 μM) condition for 10 days. Three independent transgenic lines were used in each case. Each immunoblot result is the representative of at least three repeats. **e** Phosphorylation status and protein levels of CBL1^WT^−3flag or CBL1^S201A^−3flag under low-K^+^ stress. Transgenic plants expressing similar level of CBL1^WT^−3flag or CBL1^S201A^−3flag in the *cbl1cbl9* background were grown under high-K^+^ (20 mM) condition for 4 days and then were transferred to low-K^+^ (10 μM) medium for 0, 3, or 6 days. In **b** and **c** total protein samples were subjected to regular PAGE and phostag-PAGE analyses, followed by immunoblot with CBL1 antibody, the amount of β-tubulin was determined in parallel as a loading control. **f** Quantification of CBL1^WT^−3flag and CBL1^S201A^−3flag protein abundance shown in **e**. CBL1^WT^−3flag or CBL1^S201A^−3flag protein levels were normalized to β-tubulin. The value of the starting point was set to 1. Quantitative data are shown as means ± s.e.m., *n* = 6 (biologically independent experiments). *P* value represents statistically significant differences between groups by two-way analysis of variance (ANOVA) followed by a Sidak’s multiple comparisons test.

Given that CBL1/9 proteins interact with and are phosphorylated by CIPK23 and CIPK9 in vitro^27,44,49^, and CIPK9 and CIPK23 play essential roles in regulating K^+^ uptake and homeostasis^27,29,31^, we examined whether these CIPKs are responsible for low-K^+^-enhanced CBL1/9 phosphorylation in vivo. We first monitored the phosphorylation status of CBL1/9 in wild type, *cipk23*, and *cipk9* single mutants but found no significant difference among seedlings of different genotypes (Supplementary Fig. 12a). In *cipk9/23* double mutant, however, low-K^+^-induced CBL1/9 phosphorylation was dramatically reduced (Fig. 2a), suggesting that CIPK23 and CIPK9 function redundantly in phosphorylating CBL1/9 proteins under low-K^+^ stress.

Interestingly, CIPK9/23 appeared to contribute to CBL1/9 accumulation because CBL1/9 protein abundance was much lower in *cipk9/23* double mutant than in the wild type under low-K^+^ condition (Fig. 2a). Furthermore, the kinase activity of CIPK9/23 appeared to be required for CBL1/9 accumulation under low-K^+^ stress, since low-K^+^-triggered CBL1/9 protein accumulation was clearly reduced in lines expressing the kinase-dead version of CIPKs (CIPK9^K48N^−3flag or CIPK23^K60N^−3flag) as compared to lines expressing wild-type versions (CIPK9-3flag or CIPK23-3flag) (Supplementary Fig. 9c, d).

Considering that CBL2/3 are phosphorylated by CIPK9 in vitro^49^ and CIPK9/23 were required for CBL1/9 phosphorylation in plant response to low-K^+^ stress (Fig. 2a), we speculated that low-K^+^-triggered CBL2/3 phosphorylation may also depend on CIPK9/23. Indeed, *cipk9/23* double mutants showed a drastic reduction in CBL2/3 phosphorylation status as compared to wild-type seedlings under low-K^+^ conditions. By comparing the intensity of CBL2/3 phosphorylation in *cipk9* and *cipk23* single mutants, *cipk9/23* double mutant, and wild type, we found that CIPK9 played a more dominant role over CIPK23 in phosphorylating CBL2/3 (Supplementary Fig. 12b, c). This result is in line with the previous findings that CIPK9 is more important than CIPK23 in vacuolar K^+^-remobilization^16^ and that CIPK9 preferentially phosphorylates CBL2/3 over CBL1/9 in vitro^49^. Additionally, low-K^+^-induced increase in CBL2/3 protein abundance showed a clear reduction in *cipk9/23* double mutants as compared to the wild type (Fig. 2b; Supplementary Fig. 12b), suggesting that CIPK9/23 not only mediates the phosphorylation but also facilitate the accumulation of CBL2/3 proteins under K^+^-deficiency condition.

Our data so far tightly linked CBLs phosphorylation to their stability: low-K^+^-induced phosphorylation parallels with higher protein abundance and high-K^+^-induced dephosphorylation correlates with protein degradation (Fig. 1e–h, Fig. 2a, b). Moreover, CIPKs kinase activity appeared to be indispensable for low-K^+^-induced CBLs accumulation (Supplementary Fig. 9c, d). To further confirm that phosphorylation contributes to the abundance of CBLs under low-K^+^ stress, we identified and mutated the phosphorylation sites in CBLs. Previous in vitro studies identified a conserved Ser residue within the C terminus of CBL1, CBL9, and CBL2 as the only site phosphorylated by their interacting CIPKs^31,43,44^ (Fig. 2c). To investigate whether the same site is phosphorylated in plants upon low-K^+^ stress, we used CBL1 as an example and introduced 3x flag epitope-tagged CBL1^WT^, CBL1^S201A^ into *cbl1/9* double mutant. Figure 2d showed that CBL1^S201A^ mutation abolished low-K^+^-mediated phosphorylation, confirming that Ser201 is the only phosphorylation site in CBL1 in *Arabidopsis* seedlings under low-K^+^ treatment. We next explored the relationship between CBL1 phosphorylation and protein abundance by analyzing the effect of S201A mutation on the CBL1 protein accumulation in response to K^+^ deprivation (Fig. 2e, f). Upon high- to low-K^+^ transfer, accumulation of CBL1^S201A^−3flag was reduced by more than half as compared to CBL1^WT^−3flag, confirming that the CBL1 phosphorylation contributes to its protein accumulation upon low-K^+^ stress. Despite lack of phosphorylation, abundance of CBL1^S201A^−3flag was induced by low-K^+^ albeit to a lesser extent (Fig. 2e, f), implicating other mechanism(s), in addition to phosphorylation, in controlling CBL1 stability. We investigated whether phosphorylation of CBL1 at Ser-201 impacts its function in plants under low-K^+^ stress. Consistent with the previous study^27^, *cbl1/9* double mutant plants were stunted with chlorotic leaves when grown on low-K^+^ (50 μM) medium containing 20 mM NH_4_^+^. Expression of CBL1^WT^−3flag, but not CBL1^S201A^−3flag, fully rescued the growth of *cbl1/9* double mutant (Supplementary Fig. 13). This result indicated that S201 site phosphorylation is important for the function of CBL1 in plants under low-K^+^ stress.

### Vacuolar CBLs responds to low-K^+^ more rapidly and contribute to the activation of plasma membrane CBLs

**Fig. 3:**
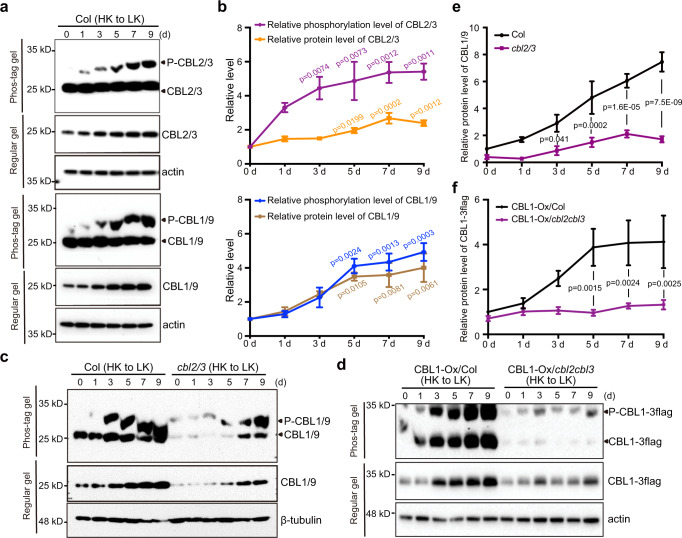
Low-K^+^-induced phosphorylation of CBL2/3 happens earlier than that of CBL1/9 and CBL2/3 contribute to the stabilization of CBL1/9 proteins. **a** Phosphorylation of CBL2/3 (upper panel) and CBL1/9 (lower panel) in Col plants upon high- to low-K^+^ transfer. Col seedlings were first grown under high-K^+^ (20 mM) condition for 6 days and then transferred to low-K^+^ (10 μM) medium for the number of days as indicated. **b** Quantification of CBL2/3 and CBL1/9 phosphorylation and protein abundance shown in (**a**). Protein abundance was normalized against β-tubulin loading control on the same Regular gel. Phosphorylated CBLs were normalized against total CBLs protein levels on the same Phos-tag gel. The value of the starting point (day 0) was set to 1. Quantitative data are shown as means ± s.e.m., *n* = 3 (biologically independent experiments). P values represent statistically significant differences between plants before and after low-K^+^ treatment, as calculated by one-way analysis of variance (ANOVA) followed by a Tukey’s multiple comparison test. **c** CBL1/9 phosphorylation and protein abundance in Col and *cbl2/3* double mutant after high- to low-K^+^ transfer. Col and *cbl2/3* seedlings were first grown under high-K^+^ (20 mM) condition for 4 days and then transferred to low-K^+^ (10 μM) medium for the number of days as indicated. **d** CBL1-3flag phosphorylation and protein abundance in the background of Col and *cbl2/3* double mutant after high- to low-K^+^ transfer. Seedlings were first grown under high-K^+^ (20 mM) condition for 4 days and then transferred to low-K^+^ (10 μM) medium for the number of days as indicated. **e** Quantification of CBL1/9 protein levels shown in **c**. CBL1/9 protein levels were normalized to β-tubulin. CBL1/9 protein level in Col at the starting point was set to 1. Quantitative data are shown as means ± s.e.m., *n* = 4 (biologically independent experiments). **f** Quantification of CBL1/9-3flag protein levels shown in **d**. CBL1-3flag protein levels were normalized to actin. CBL1-3flag protein level in Col at the starting point was set to 1. Quantitative data are shown as means ± s.e.m., n = 6 (biologically independent experiments). In **e** and **f**, *P* values represent statistically significant differences between groups as calculated by two-way analysis of variance (ANOVA) followed by a Sidak’s multiple comparisons test. In **a**, **c**, **d**, total protein samples were subjected to regular PAGE and phostag-PAGE analyses. CBL1/9 and CBL2/3 proteins were analyzed using CBL1 antibody and CBL3 antibody, respectively. CBL1-3flag proteins were analyzed using flag antibody. β-tubulin or actin was used as a loading control.

Previous studies show that, at the early stage of plant responses to K^+^ shortage, vacuolar K^+^ concentration drops dramatically to maintain steady cytoplasmic K^+^ levels^6,10^. In addition, the vacuolar CBL-CIPK pathway is more important in response to short-term K^+^-deficiency^16^. We, therefore, hypothesized that, when facing low-K^+^ availability, activation of vacuolar K^+^ remobilization by VM-CBL2/3-CIPK pathway may serve as a primary mechanism to supplement cytoplasmic K^+^^16^. As a result, VM-CBL-CIPK pathway may be activated first in response to low-K^+^ stress. To test this hypothesis, we grew wild-type seedlings on the high-K^+^ medium for 6 days and transferred them to low-K^+^ medium to monitor the time course of phosphorylation of CBL1/9 and CBL2/3 in parallel. As shown in Fig. 3a, b, the phosphorylation level of CBL2/3 proteins was elevated by 4.5-fold on the third day upon K^+^ starvation treatment, whereas CBL1/9 phosphorylation level did not show a significant increase until the fifth day, supporting the notion that VM-CBL2/3 activation happens before PM-CBL1/9 and vacuolar K^+^ remobilization is the primary response to low-K^+^ stress.

To further explore the relationship between the two CBL-CIPK pathways, we examined whether disruption of one pathway had positive or negative impact on the activation of the other pathway in response to low-K^+^ stress. We found that low-K^+^-induced increase in CBL2/3 protein abundance and phosphorylation remained the same in the wild type and *cbl1/9* double mutant (Supplementary Fig. 14), indicating that PM-CBL1/9 proteins are not essential for the activation of VM-CBL2/3-CIPK pathway. In contrast, protein abundance of CBL1/9 was severely reduced in *cbl2/3* double mutant as compared to the wild type (Fig. 3c, e), suggesting that CBL2/3 are indispensable for the up-regulation of CBL1/9 protein accumulation under low K conditions. To further confirm this observation, we crossed UBQ10:CBL1-3flag transgenic line into the *cbl2/3* background and compared the protein level of CBL1-3flag in *cbl2/3* and wild-type backgrounds. Like the native CBL1, CBL1-3flag protein level remained very low in the *cbl2/3* during a 9-day low-K^+^ treatment (Fig. 3d, f). Because a constitutive promoter was utilized to drive transcription of CBL1-3flag protein, changes in low-K^+^-induced CBL1/9 protein accumulation, as regulated by CBL2/3, occurred at a post-translational level. Taken together, these results support the conclusion that VM-CBL2/3-CIPK pathway is the first responder to low-K^+^ stress and, further, the vacuolar pathway positively impacts the activation of the PM-CBL1/9-CIPK pathway.

### Vacuolar CBL-CIPK pathway is negatively regulated by PP2C phosphatases

If the CBLs are phosphorylated by CIPKs and become more stable in response to low-K^+^ stress, they may be dephosphorylated and destabilized upon K^+^ replenishment. The such reversible regulatory mechanism may hold the key to enabling plant adaptation to the changes in K^+^ nutrient status. To address this mechanism further, we sought to identify the phosphatases required for the dephosphorylation of CBLs in response to high-K^+^. We previously showed that multiple PP2C (Protein phosphatase type 2C) Group A members interact with CIPKs and CBLs in yeast^23,50^. We hypothesized that CBLs, CIPKs, and PP2Cs may form alternate complexes in regulating the kinase activity of CIPKs and the phosphorylation levels of CBLs. To test this hypothesis, we performed in vitro kinase assay and tested the effect of four group A PP2Cs, HAB1/ABI1/ABI2/PP2CA, on the phosphorylation of CIPK9 and/or CBL2/3.

**Fig. 4:**
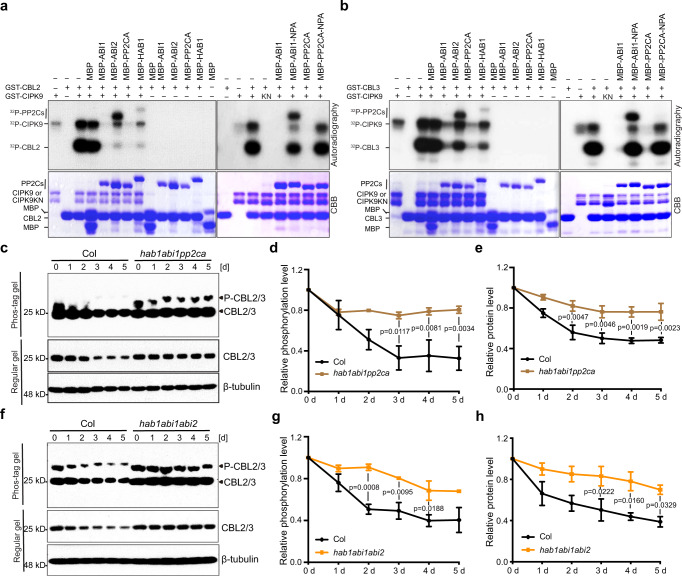
HAB1/ABI1/ABI2/PP2CA phosphatases are responsible for CBL2/3 dephosphorylation upon K^+^-repletion. **a, b** In vitro kinase assays using either GST-CBL2 (**a**) or GST-CBL3 (**b**) as substrate for CIPK9 or kinase-dead CIPK9-KN mutant in the presence or absence of different PP2C members. Phosphorylated proteins were separated by SDS-PAGE gels and detected by autoradiography (upper panel). Total proteins were quantified in Coomassie-stained SDS-PAGE gels (bottom panel). The experiments were repeated three times and one representative image is shown. **c** Phosphorylation and protein levels of CBL2/3 in Col and *hab1abi1pp2ca* triple mutant after low- to high-K^+^ transfer. **d**, **e** Quantification of CBL2/3 phosphorylation level (**d**) and protein level (**e**) shown in **c**. **f** Phosphorylation and protein levels of CBL2/3 in Col and *hab1abi1abi2* triple mutant after low- to high-K^+^ transfer. **g, h** Quantification of the CBL2/3 phosphorylation level (**g**) and protein level (**h**) shown in **f**. Col, *hab1abi1pp2ca*, and *hab1abi1abi2* triple mutant seedlings were first grown under low-K^+^ (10 μM) for 5 days and then transferred to high-K^+^ (20 mM) medium for the indicated time (d, days). In **d** and **g**, Levels of phosphorylated CBL2/3 were normalized against total CBL2/3 protein levels on Phos-tag gel. The value of the starting point (0 d) was set to 1. In **e** and **h**, CBL2/3 protein abundance was normalized against β-tubulin loading control on the same Regular gel. The value of the starting point (day 0) was set to 1. In **d**, **e**, **g**, **h**, Data are shown as means ± s.e.m., *n* = 3 (biologically independent experiments). P values represent statistically significant differences between groups by two-way analysis of variance (ANOVA) followed by a Sidak’s multiple comparisons test. CBL2/3 abundance was analyzed using CBL3 antibody. β-tubulin was used as a loading control.

As shown in Fig. 4a, b, the recombinant CIPK9, but not the dead kinase CIPK9^K48N^, showed autophosphorylation activity and phosphorylated CBL2 and CBL3. The kinase activity of CIPK9 was dramatically enhanced by CBL2 or CBL3, consistent with a previous study^49^. When ABI1, ABI2, HAB1, or PP2CA was added to the reaction, both autophosphorylation and CBL2/3 transphosphorylation were effectively abolished (Fig. 4a, b). When the phosphatase activity was disrupted by a mutation in these PP2Cs, however, they became ineffective in blocking the CIPK and CBL phosphorylation. For example, D177A mutation in ABI1 or D142A mutation in PP2CA, which abrogates Mn^2+^ binding and impairs phosphatase activity^51,52^, eliminated the inhibitory effect of ABI1 and PP2CA against CIPK9 and CBL2/3 phosphorylation in our assays (Fig. 4a, b). Interestingly, CIPK9 phosphorylated MBP-ABI2 and MBP-HAB1 proteins but not MBP-ABI1 and MBP-PP2CA, implying that CIPK9 may utilize ABI2 and HAB1 as substrates thereby regulating their phosphatase activities.

To further investigate whether ABI1, ABI2, PP2CA and HAB1 dephosphorylate CBL2/3 *in planta*, we monitored CBL2/3 dephosphorylation in two triple mutants impaired in the above four phosphatases, *hab1abi1pp2ca* and *hab1abi1abi2*. In wild type seedlings, CBL2/3 was substantially dephosphorylated 3 days after transfer from low- to high-K^+^ (Fig. 4c, d, f, g). In striking contrast, CBL2/3 remained phosphorylated in *hab1abi1pp2ca* and *hab1abi1abi2* triple mutants through 4–5 days after K^+^-replenishment. Surprisingly, the PM-localized CBL1/9 proteins underwent dephosphorylation to the same extent in the triple mutants as in the wild type (Supplementary Fig. 15). These observations identified HAB1/ABI1/ABI2/PP2CA as essential components required for the dephosphorylation of CBL2/3, but not for CBL1/9, in response to sufficient-K^+^ levels despite the fact that same CIPKs are responsible for phosphorylating both CBL1/9 and CBL2/3 (Fig. 2a, b). Consistent with the phosphorylation status during low- to high-K^+^ transfer, CBL2/3 protein abundance was significantly reduced in the wild type but not in *hab1abi1pp2ca* and *hab1abi1abi2* triple mutants (Fig. 4c, e, f, h), suggesting that these phosphatases are also involved in the regulation of degradation of CBL2/3 under high-K^+^ condition. Given that all of these four PP2C group A members act as key negative regulators of ABA responses^53^, and that the biosynthesis of ABA in both leaves and roots showed an increase after K^+^ starvation^54^, we hypothesized that high-K^+^ activates HAB1/ABI1/ABI2/PP2CA phosphatases by down-regulating ABA level, leading to CBL2/3 dephosphorylation and degradation. This possibility was supported by the observation that high-K^+^-induced dephosphorylation and degradation of CBL2/3 was substantially reduced by the addition of ABA to the medium (Supplementary Fig. 16).

### The ABA signaling PP2Cs are negative regulators of low-K response

**Fig. 5:**
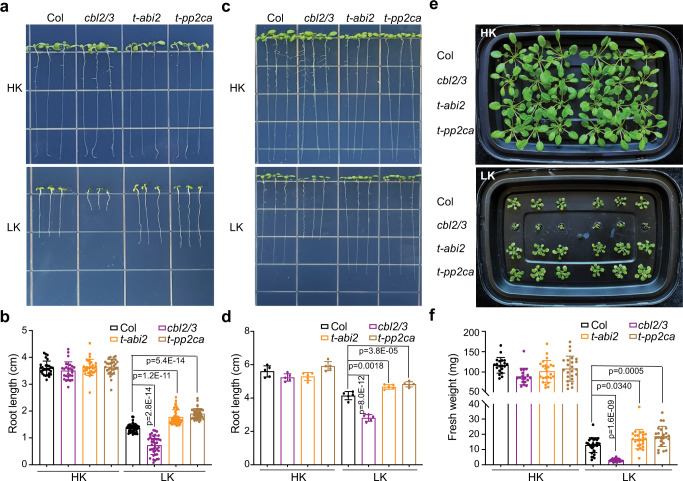
*hab1abi1pp2ca* and *hab1abi1abi2* triple mutants show tolerant phenotype under low-K^+^ stress. **a** Seed germination of wild type (Col-0), *cbl2/3* double mutant, *hab1abi1pp2ca* and *hab1abi1abi2* triple mutants under high- and low-K^+^ conditions. Representative images show 7-day-old seedlings after direct germination on the medium containing high-K^+^ (20 mM) condition or low-K^+^ (5 μM) condition. (**b**) Measurement of root length at the end of the assay as shown in (**a**). **c** Representative images of wild type (Col), *cbl2/3* double mutant, *hab1abi1pp2ca* and *hab1abi1abi2* triple mutants under high- or low-K^+^ in post-germination assays. The seedlings were grown under sufficient-K^+^ (20 mM) condition for 4 days, followed by a transfer to high-K^+^ (20 mM) or low-K^+^ (5 μM) conditions and grown for another 5 days. **d** Measurement of root length at the end of the assay as shown in (**c**). **e** Growth phenotype of 5-week-old wild type plants, *cbl2/3* double mutant, *hab1abi1pp2ca* and *hab1abi1abi2* triple mutants in hydroponic solutions containing high- or low-K^+^. 7-day-old seedlings germinated on MS plates were transferred to hydroponic solutions containing high-K^+^ (20 mM) or low-K^+^ (1 μM) for another 4 weeks. Photographs were taken at the end of the assay. **f** Measurement of the fresh weight of 5-week-old plants in hydroponic solutions as shown in **e**. Data in **b**, **d,** and **f**, are shown as mean ± SD, *n* = 3 (biologically independent experiments). Statistical analyses between groups were performed by one-way analysis of variance (ANOVA) followed by a Tukey’s multiple comparison test.

The HAB1/ABI1/ABI2/PP2CA phosphatases countered CBL2/3-CIPK9 phosphorylation (Fig. 4), which may negatively regulate CBL-CIPK activity in response to low-K^+^ stress. We examined *hab1abi1pp2ca* and *hab1abi1abi2* mutants under low-K^+^ stress and found that they displayed an opposite phenotype to the *cbl2/3* double mutant. At 20 mM K^+^ concentration (equivalent to the concentration of K^+^ in MS medium), there was no discernible difference in the seedlings of the mutants. Under a low-K^+^ condition (5 μM K^+^), *cbl2/3* double mutant showed strong growth retardation as exemplified by shorter roots during early seedling development (Fig. 5a–d), consistent with the finding in our previous study^55^. In contrast, both *hab1abi1pp2ca* and *hab1abi1abi2* triple mutants were significantly more tolerant to low-K^+^ condition, showing longer primary roots than both *cbl2 cbl3* double mutant and the wild type (Fig. 5a–d).

To further address the role of HAB1/ABI1/ABI2/PP2CA in low-K^+^ stress during an extended growth period, we cultured the seedlings hydroponically and examined their phenotypes for 4 weeks. As shown in Figure 5e, f, *hab1abi1pp2ca* and *hab1abi1abi2* triple mutants as well as *cbl2/3* double mutant did not show any significant difference from the wild-type plants under high-K^+^ condition. When grown in the low-K^+^ solution (1 μM), *cbl2/3* mutant seedlings were severely stunted with leaf necrosis as previously reported^55^. In contrast, *hab1abi1pp2ca*, *hab1abi1abi2* mutants grew significantly better than wild-type plants (Fig. 5e, f), suggesting that HAB1/ABI1/ABI2/PP2CA phosphatases play a negative role in plant growth under low-K^+^ stress.

**Fig. 6:**
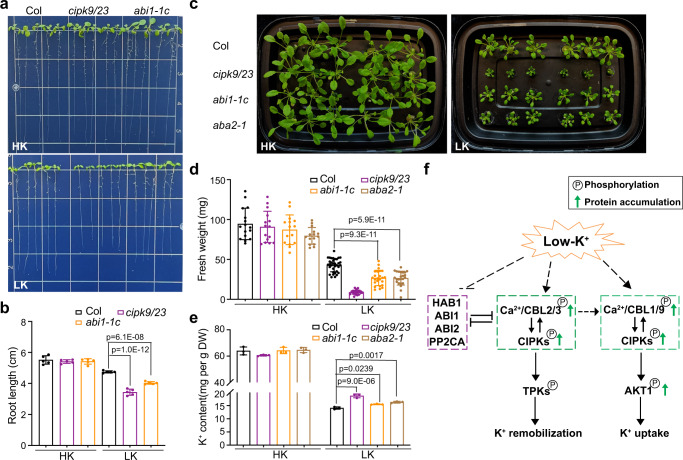
*abi1-1c* and *aba2-1* mutant plants are hypersensitive to external K^+^ deficiency. **a** Representative images of wild type (Col-0), *cipk9/23* double mutant, *abi1-1c* mutants under high- or low-K^+^ in post-germination assays. The seedlings were grown under sufficient-K^+^ (20 mM) conditions for 4 days, followed by a transfer to high-K^+^ (20 mM) or low-K^+^ (5 μM) condition and grown for another 5 days. **b** Measurement of root length at the end of the assay as shown in **a**. **c** Growth phenotype of 5-week-old plants of Col-0, *cipk9/23* double mutant, *abi1-1c* and *aba2-1* mutants in hydroponic solutions containing high- or low-K^+^. 7-day-old seedlings germinated on MS plates were transferred to hydroponic solutions containing high-K^+^ (20 mM) or low-K^+^ (10 μM) for another 4 weeks. Photographs were taken at the end of the assay. **d** Measurement of the fresh weight of plants in hydroponic solutions as shown in **d**. **e** Quantification of K content in various plant materials was measured at the end of the K^+^-starvation assay as shown in **c**. Data in **b**, **d**, and **e** are shown as mean ± SD, *n* = 3 (biologically independent experiments). Statistical analysis between groups were performed by one-way analysis of variance (ANOVA) followed by a Tukey’s multiple comparison test. **f** A working model depicting the regulation of dual CBL-CIPK modules in plant responses to changes in K^+^ status. Low-K^+^ stress enhances protein abundance and phosphorylation of Ca^2+^ sensors (vacuolar CBL2/3, plasma membrane CBL1/9) and their CIPK partners. The dual CBL-CIPK modules activate target transporters such as TPK1 and AKT1to increase K remobilization and uptake, which ultimately enable plants to adapt to low-K^+^ conditions. Upon K^+^-repletion, dual CBL-CIPK modules are deactivated through dephosphorylation and protein degradation. The PP2C family phosphatases, including HAB1/ABI1/ABI2/PP2CA, specifically deactivate the vacuolar CBL-CIPK module.

To further assess the function of HAB1/ABI1/ABI2/PP2CA phosphatases in plant response to low-K^+^ stress, we evaluated the growth of the dominant gain-of-function mutant *abi1-1c* in which the mutated ABI1 becomes constitutively active^56^. Our results showed that *abi1-1c* seedlings were hypersensitive to low-K^+^ condition as indicated by shorter roots, although the phenotype was not as strong as in the *cipk9/23* double mutant (Fig. 6a, b). In the hydroponic solutions containing low-K^+^ (10 μM), the fresh weight of *abi1-1c* plants was reduced to 60% of that of the wild type (Fig. 6c, d). In addition, ABA-deficient mutant *aba2-1*^57^ was also more stunted than wild-type plants under low-K^+^ conditions (Fig. 6c, d). Moreover, *abi1-1c* and *aba2-1* plants grown under low-K^+^ showed a significantly higher K content than the wild type, in line with the elevated K content in the *cipk9/23* double mutant (Fig. 6e). Taken together, these data support the conclusion that HAB1/ABI1/ABI2/PP2CA phosphatases play a negative role in plant response and adaptation to low-K^+^ stress.

## Discussion

To respond and adapt to low-K^+^ environments, plants have evolved an intricate mechanism involving dual CBL-CIPK calcium signaling pathways. The PM-CBL1/9-CIPK pathway activates the AKT1 channel and other transporters in the roots to boost K^+^ uptake, and the VM-CBL2/3-CIPK pathway enhances vacuolar K^+^-remobilization to support cytoplasmic metabolism. Although the CBL-CIPK-K^+^ transport nexus has been well established, the early events that link K^+^ status to activation of CBL-CIPK proteins remains unclear. In this study, we identified post-translational modifications of PM-CBL1/9-CIPKs-AKT1 and VM-CBL2/3-CIPK9/23-TPK1 proteins in response to changing external K^+^ levels, adding unique insights to the molecular processes responsible for nutrient sensing in plants.

In the context of mineral nutrient transport in plants, studies have shown that abundance of transporter proteins can respond to substrate concentration. For example, phosphate (P) uptake transporters of the PHT1 family are induced by low-P at the transcriptional level and degraded in response to P repletion^58,59^. In the case of K^+^ sensing, we have shown here that not only the transporter proteins (e.g., AKT1) but also the components in the low-K^+^ response signaling pathways (i.e., CBLs and CIPKs) respond to K^+^ status by altering their protein abundance (Fig. 1). In addition, the regulation of protein levels occurs at the post-translational level for all components, calling for future effort to identify the enzymes and regulators that connect nutrient availability to protein stability. In fact, our effort here indicates that one mechanism for protein stability control, at least for CBL calcium sensors, involves phosphorylation by their partner kinases (Fig. 2). Although previous studies showed that the CBLs can be phosphorylated in vitro by their partner CIPKs, functional relevance of such phosphorylation is proposed to enhance interaction between CBLs and CIPKs in yeast and protoplast transient expression system or to play a role in activating AKT1 in *Xenopus* Oocytes^43,44,60^. In this study, we identified specific CIPKs responsible for CBL phosphorylation *in planta* and provided a link between CBL phosphorylation and control of the protein stability in response to changing K^+^ status. Because the abundance of downstream kinases (CIPKs) and transporters (e.g., AKT1) are also tightly associated with their phosphorylation status, we propose that the “CBL-CIPK-transporter” pathway represents a phosphorylation-dependent protein stabilization and activation cascade (Fig. 6f). Investigating the mechanism underlying regulated protein stability in response to K status and how different CIPK members may differentially regulate different CBLs will be a major direction to focus on in the future.

For further understanding of phosphorylation-dependent protein stability control, we identified the kinases and phosphatases that contribute to the reversible phosphorylation of the CBL-type Ca^2+^ sensors. It is particularly interesting to find that the CBL-phosphorylating kinases (CIPKs) are activated by the low-K^+^ stress and, in contrast, the PP2C phosphatases function in response to low- to high-K^+^ switch. Furthermore, it is significant to reveal that the CIPKs responsible for low-K^+^ response phosphorylated both the PM-CBLs and VM-CBLs (Fig. 2a, b), whereas the HAB1/ABI1/ABI2/PP2CA phosphatases involved in high-K^+^ response specifically act on the VM-CBLs (Fig. 4, Supplementary Fig. 15). Such findings clearly indicate that high-K^+^-induced dephosphorylation are pathway-specific and does not simply represent a reverse mode of low-K^+^-induced phosphorylation. Taken together with previous finding that the high K^+^-responsive PP2Cs (HAB1/ABI1/ABI2/PP2CA) have been shown to be critical regulators of ABA responses^53^, we expect that K^+^-nutrient sensing may crosstalk to ABA signaling through these and possibly other components. The finding that the addition of ABA to the high-K^+^ medium blocked the VM-CBL2/3 dephosphorylation and ABA deficient mutant *aba2-1* showed a similar hypersensitive phenotype to *cbl2/3* and *cipk9/23* mutants supported this notion (Supplementary Fig. 16, Fig. 6c–e). Considering that these PP2C members can physically interact with both CIPKs and CBLs^23,50^, and that HAB1/ABI1/ABI2/PP2CA repress the auto-phosphorylation of CIPK9 and CBL2/3 transphosphorylation (Fig. 4a, b), we proposed that HAB1/ABI1/ABI2/PP2CA phosphatases may control the phosphorylation levels of CBL2/3 by at least three possible mechanisms: by interacting and dephosphorylating CBL2/3 directly, by dephosphorylating and inhibiting CIPK9/23 kinase activity, and/or by sequestering CIPK9/23 proteins from binding to CBL2/3. Future work is thus expected to resolve these possibilities and to identify other early events in sensing K^+^ status in plants.

Another significant finding in this work is the relationship between the dual CBL-CIPK pathways in response to low-K^+^ stress. Our recent study indicated that VM-CBL2/3-CIPK pathway is more critical than the PM-CBL1/9-CIPK pathway because *cbl2cbl3* double mutant showed severe growth inhibition in a broad range of external K^+^ regimes whereas the *cbl1cbl9* double mutant displayed much less defect under the same conditions^16^. This is consistent with our results in this study that the activity of VM-CBL2/3-CIPK pathway is more sensitive to K^+^ deficiency and is activated earlier than PM-CBL1/9-CIPK pathway during high- to low-K^+^ transfer (Fig. 3a, b). These results further support the notion that VM-CBL2/3-CIPK pathway for K^+^ remobilization may serve as a primary mechanism for plants to respond and adapt to K^+^-deficiency^16^. Along this line, we also found that, although CBL2/3 and CBL1/9 are spatially separated in the cell, VM-CBL2/3 are essential for the stabilization of CBL1/9 proteins in response to low-K^+^ stress (Fig. 3c–f). Concerning the molecular link that enables such coordination between VM-CBL2/3-CIPK and PM-CBL1/9-CIPK pathways, we hypothesize that the partner kinases shared by CBL2/3 and CBL1/9, i.e., CIPK9/23, may serve as the “bridge” of the dual pathways. When plants face low-K^+^ stress, early signals, such as Ca^2+^ spikes, may first activate CBL2/3 that preferentially recruit CIPK9/23 kinases to the vacuolar membrane to phosphorylate and stabilize CBL2/3. Stable CBL-CIPK complexes phosphorylate and activate their target transporters such as TPKs to retrieve K^+^ from the vacuolar store. With prolonged K^+^ deficiency, the PM-CBL1/9 recruit the hyperactive CIPKs that may shuttle between VM and PM to form CBL-CIPK complexes at the plasma membrane where CIPKs phosphorylate CBL1/9 and K^+^ transporters, e.g., AKT1, boosting K^+^ uptake from K^+^-limited environments. Future work should be directed to monitoring the time course of low-K^+^ generated Ca^2+^ signature and its correlation with the sequential activation of VM-CBL-CIPK and PM-CBL-CIPK modules, as well as dissecting the possible “CIPK shuttling” mechanism between the VM and PM. Additionally, it would be interesting to explore the molecular toolkit for the production of Ca^2+^ signals that activate CBL2/3 and CBL1/9 in response to low-K^+^ stress.

## Methods

### Plant materials and growth conditions

All the wild-type, mutant and transgenic *Arabidopsis* lines used in this study are Columbia (Col-0) ecotype. Detailed information on T-DNA insertion mutants used in this study is as follows: *cbl1*^27^, *cbl9*^27^, *cbl1/9* (*cbl1cbl9*)^27^, *cbl2/3*(*cbl2cbl3*)^16^*, cipk23-1* (SALK_032341)^37^, *cipk9-1* (SAIL_252_F06)^61^, *cipk9/23* (*cipk9cikp23*)^16^, *hab1abi1pp2ca*^53^, *hab1abi1abi2*^53^*, akt1* (SALK_071803)^27^, *abi1-1c*^56^, *aba2-1*^57^.

### Generation of transgenic plants

To generate UBQ10: CBL1^WT^−3flag transgenic plants, CBL1 CDS was amplified by PCR and was fused in-frame into triple-Flag in a pCambia1300-derived plasmid, followed by agrobacterium-mediated transformation into *Arabidopsis cbl1/9* double mutant. UBQ10: CBL1^S201A^−3flag construct was generated by site-directed mutagenesis using UBQ10: CBL1^WT^−3flag as template and Phusion High-Fidelity DNA polymerase (NEB) and then transformed into *cbl1/9* double mutant. UBQ10: CIPK9-3flag, UBQ10: CIPK23-3flag, UBQ10: CIPK9^K48N^−3flag, and UBQ10: CIPK23^K60N^−3flag constructs were built using the same vector as for CBL1 constructs and then transformed into *cipk9/23* double mutant, respectively. The UBQ10: AKT1-3flag was constructed using the same vector above and transformed into *akt1* mutant. The primers are listed in Supplementary Table 1.

### Growth conditions for *Arabidopsis* seedlings under different K^+^ regimes

All seeds were surface sterilized with 10% bleach for 10 min, washed three times with water and sown on the growth medium solidified with 0.8% (w/v) BD BBL^TM^ select agar. The recipe of the growth medium was modified from MS medium with a reduced level of NH_4_^+^ unless indicated otherwise, which contained the following components: 3 mM Ca (NO_3_)_2_, 1.25 mM NH_4_H_2_PO_4_, 1.5 mM MgSO_4_, 1× Murashige and Skoog (MS) micronutrients (contain 5 μM K^+^), and 1% (w/v) sucrose. The pH of the medium was adjusted to 5.8 using NaOH. The final K^+^ concentration in the medium was adjusted by adding KCl as the K^+^ source. For the germination phenotyping assay, seedings were germinated on modified MS medium shown above with different concentrations of K^+^ and incubated at 4 °C for 4 d for stratification, then were transferred to a growth chamber with 80 μmol m^−2^ s^−1^ light intensity with a 12 h light/12 h dark photoperiod for the indicated days. For the post-germination phenotyping assay, seeds were germinated on modified MS medium containing 20 mM K^+^ and grown for 4 days. The seedlings were then transferred onto various agarose-solidified modified MS medium (3 mM Ca (NO_3_)_2_, 1.25 mM NH_4_H_2_PO_4_, 1.5 mM MgSO_4_, 1× Murashige, and Skoog (MS) micronutrients, and 1% (w/v) sucrose, pH5.8) supplemented with different concentrations of K^+^ for subsequent growth under 80 μmol m^−2^ s^−1^ light intensity with a 12 h light/12 h dark photoperiod. At the end of assay, the root length of seedlings was measured by Image J software. For phenotypic assay in the hydroponics, seeds were germinated on MS medium and grown for 7 days. The seedlings were then transferred to the liquid solution containing 1.4 mM Ca(NO_3_)_2_, 0.1 mM Ca(H_2_PO_4_)_2_, 0.125 mM MgSO_4_, 0.025 mM MgCl_2_, as well as 1/6 strength of MS minor salts and supplemented with different concentrations of KCl. All the hydroponic solutions for plant growth were replaced with fresh ones twice a week.

### RNA isolation and quantitative real-time PCR analysis

Total RNA was extracted from plant samples using the TRIZOL reagent (Invitrogen). After being treated with DNase I (Invitrogen) to remove DNA contamination, cDNA was synthesized using SuperScript II reverse transcriptase kit (Invitrogen). The quantitative real-time PCR analysis was performed on the DNA Engine Opticon System (MJ Research) using the SYBR Green Realtime PCR Master Mix (Bio-Rad). All experiments were performed using three biological replicates, and actin served as an internal standard. The relative expression of each gene was calculated using ΔΔCT method (2^-ΔΔ*CT*^)^62^. Each experiment was repeated with three different batches of samples and RT-PCR reactions were performed with three technical replicates for each sample. The primers used in quantitative real-time PCR are listed in Supplementary Table 1.

### Protein extraction and immunoblots

For total protein extraction, *Arabidopsis* seedlings were grounded in the presence of liquid nitrogen to a fine powder and extracted with 2× SDS sample buffer (100 mM Tris-Cl, pH6.8, 4% SDS, 0.2% bromophenol blue, 20% glycerol and freshly added 10% β-mercaptoethanol). Aliquots of denatured total protein were separated by 12% SDS-PAGE and transferred to PVDF membrane. For the detection of phosphorylated CBL proteins, the total protein was separated by 10% SDS-PAGE with 15 μM Phos-tag (AAL-107, WAKO pure chemical industries, Ltd) and transferred to PVDF membrane. For immunoblot analyses, anti-CBL1, anti-CBL3^42^, anti-β-tubulin (Santa Cruz Biotechnology, SC-166729), anti-GAPDH (PHYTOAB, PHY0303A), anti-actin (PHYTOAB, PHY0001), anti-Flag (Sigma-Aldrich, A8592-2MG) were used as primary antibodies. The anti-CBL1 rabbit polyclonal antibody was made using recombinant CBL1 protein purified from E coli as antigen by Cocalico company (in PA). Each experiment was repeated at least three times, and one representative result was shown. Quantification of immunoblots was done using Image J software.

### Phosphatase treatment

*Arabidopsis* seedlings were grounded in the presence of liquid nitrogen to fine powder and extracted with buffer containing 50 mM HEPES (NaOH, pH 7.5), 150 mM NaCl, 50 mM β-glycerophosphate, 2 mM DTT, 1% Triton X-100 and 10% glycerol, with EDTA-free protease inhibitors (Roche). After centrifugation for 10 min at 20,000 g, the supernatant was isolated and used as protein samples for Phostag gel analysis. For dephosphorylation of CBL1/9 and CBL2/3, 50 μL supernatant was incubated with 1 μL λ-PPase (NEB) and 5 μL 10 mM MnCl_2_ under 30 °C for the indicated times. For dephosphorylation of CIPK9-3Flag, CIPK23-3Flag, AKT1-3Flag proteins, the supernatant was incubated with 10 μL prewashed anti-Flag M2 agarose beads (Sigma-Aldrich) for 1 h at 4 °C on a roller shaker. The beads were then washed three times with the extraction buffer described above. The protein bound beads were incubated with 1 μL λ-PPase (NEB) and 5 μL 10 mM MnCl_2_ under 30 °C for 30 min. The dephosphorylation reaction was stopped by adding 2× SDS loading buffer and boiled for 10 min.

### In vitro kinase assay

CIPK9, CBL2 and CBL3 were cloned in pGEX4T-1 vector and expressed in *E.coli* as a GST(glutathione S-transferase)-tag fusion protein, ABI1, ABI2, PP2CA and HAB1 were cloned in pMAL-c2X vector as a MBP (maltose-binding protein)-tag fusion protein. All MBP- and GST-fused proteins were purified according to standard instructions. For in vitro phosphorylation, 0.5–2.0 mg of purified proteins was incubated in kinase reaction buffer containing 20 mM Tris (pH 7.2), 2.5 mM MnCl_2_, 0.5 mM CaCl_2_, 1 mM DTT, 10 mM ATP and 2 μCi ^32^γP at 30 °C for 30 min and terminated by 5× SDS–PAGE loading buffer. The samples were subsequently analyzed using a 12% SDS-PAGE gel, followed by Coomassie staining and autoradiography. Coomassie staining was used to verify the quality of samples and loading consistency.

### K content

To measure the K content, plant roots and shoots were harvested separately at the end of each phenotypic assay and surface-washed with double-distilled water for 30 s. The samples were then thoroughly dried in the oven at 99 °C. The dry tissues were grounded in a mortar, collected into a 15 ml tube, and dissolved with 1 ml ultrapure HNO_3_ (Sigma-Aldrich). The tubes were incubated in a water bath at 99 °C for 4 h. Digested samples were diluted with double-distilled water and the K concentration in the solution were determined by inductively coupled plasma optical emission spectroscopy (ICP-OES; PerkinElmer).

### Total chlorophyll content

Six seedlings were collected from each group and grounded into a fine powder in liquid nitrogen. Total chlorophyll was extracted by dissolving the tissue powder in 1 ml of 80% acetone and incubated in a shaking water bath overnight in darkness. Chlorophyll content was measured spectroscopically and calculated using the equation: chlorophyll_a + b_ =  7.15 × OD_660_  +  18.71 × OD_647_.

### Reporting summary

Further information on research design is available in the Nature Portfolio Reporting Summary linked to this article.

## Supplementary information

Supplementary Information

Reporting Summary

## Data Availability

All data generated and analyzed in this study are available within the article and its Supplementary Information files. The data are available from the corresponding author upon reasonable request. Source data are provided with this paper.

## Source data

Source Data
