# Supplementary material for: Potassium nutrient status drives posttranslational regulation of a low-K response network in Arabidopsis
Source: Nat Commun. 2023 Jan 23;14:360. doi: 10.1038/s41467-023-35906-5 (PMC9870859; doi:10.1038/s41467-023-35906-5)
Supplement: Supplementary file 1 — Supplementary Information [file 41467_2023_35906_MOESM1_ESM.pdf]

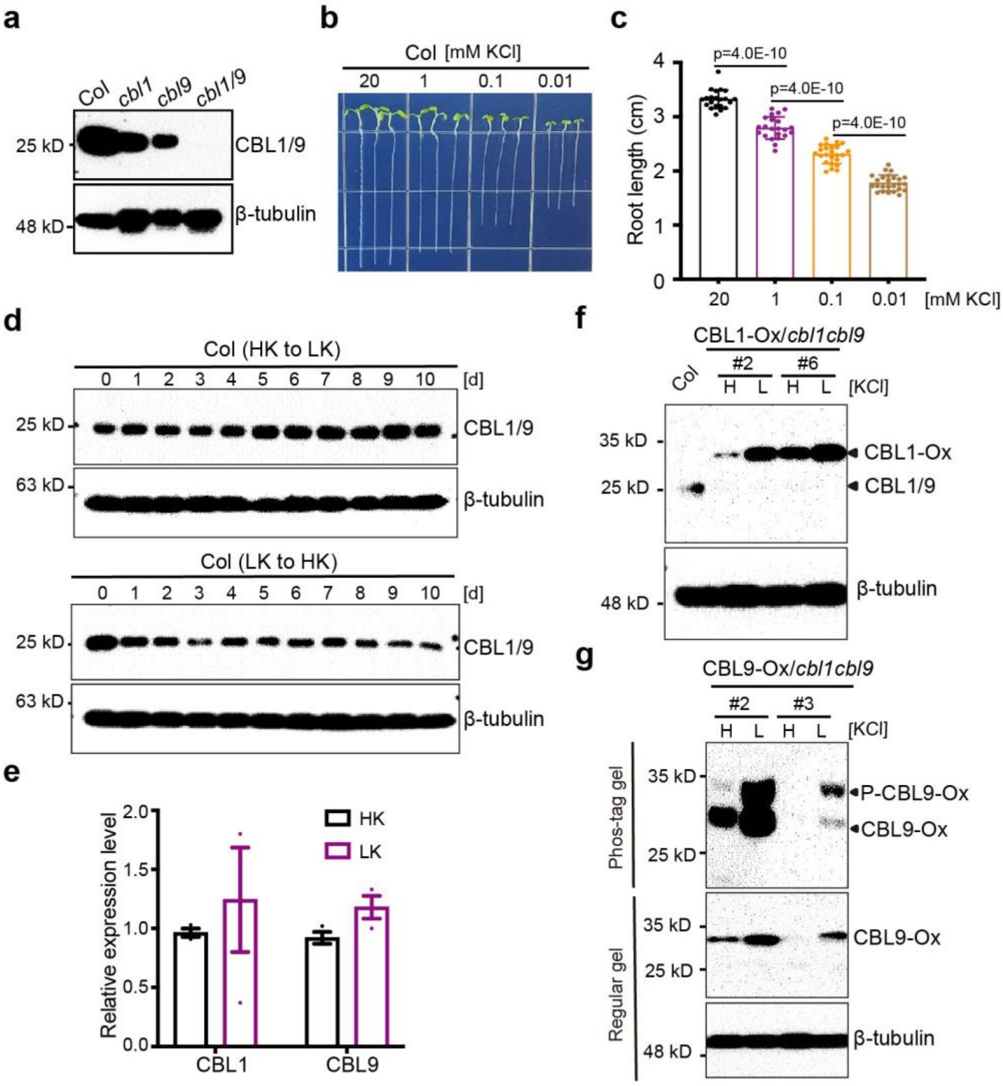

Supplementary Figure 1. K^+ status controls CBL1/9 protein abundance. (**a**) Immunoblot analysis of CBL1/9 abundance in the wild type (Col), *cbl1*, *cbl9*, and *cbl1/9* (*cbl1cbl9*) mutants. (**b**) Representative images of Col plants grown under different external K^+ concentrations (20, 1, 0.1, 0.01 mM) for 7 days. (**c**) Measurement of seedlings root length at the end of the seed germination assay as shown in **b**. Independent experiments were repeated for three times. Statistical analyses between groups were performed by one-way ANOVA followed by a Turkey's multiple comparison test. (**d**) Changes in CBL1/9 protein abundance in Col seedlings after high- to low-K^+ transfer or vice versa. During high- to low-K^+ transfer, Col seedlings were first grown under high-K^+ (20 mM) for 7 days and then transferred to low-K^+ (10 μM) for the indicated time (days) before sampling and analysis by immunoblots using the CBL1 antibody. During low- to high-K^+ transfer, Col seedlings were first grown under low-K^+ for 7 days and then transferred to high-K^+ (20 mM) for the indicated time (days) before analyzed as described above. (**e**) Quantitative RT-PCR analysis of CBL1 and CBL9 transcripts under high- or low-K^+ condition. Col seedlings were grown on the medium containing high-K^+ (20 mM) or low-K^+ (10 μM) for 7 days. The relative expression of each gene was double normalized against the expression level of ACTIN2 and the expression level from plants grown under high-K^+ condition. Data are shown as mean ± s.e.m, n=3 (biologically independent experiments). (**f**) The protein level of CBL1 and CBL1-3flag in Col, UBQ10: CBL1-

3flag/*cbl1cbl9* seedlings grown under high-K^+ (20 mM) or low-K^+ (10 μM) for 7 days. (**g**) The protein level and phosphorylation status of CBL9-3flag in UBQ10: CBL9-3flag/*cbl1cbl9* seedlings grown under high-K^+ (20 mM) or low-K^+ (10 μM) for 7 days. CBL1/9, CBL1-3flag, CBL9-3flag proteins were detected using the CBL1 antibody. β-tubulin was used as a loading control. Each immunoblot result is the representative of at least three repeats.

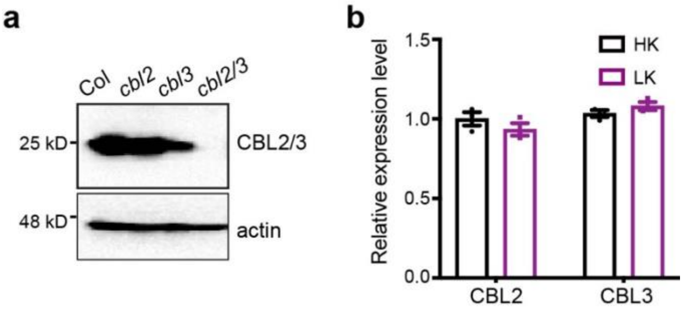

Supplementary Figure 2. (a) Immunoblot analysis of CBL2/3 abundance in the wild type (Col), *cbl2*, *cbl3*, and *cbl2/3* (*cbl2cbl3*) mutants. (**b**) Quantitative RT-PCR analysis of CBL2 and CBL3 transcripts under high- or low-K^+ condition. Col seedlings were grown on the medium containing high-K^+ (20 mM) or low-K^+ (10 μM) for 7 days. The relative expression of each gene was double normalized against the expression level of ACTIN2 and the expression level in plants grown under high-K^+ condition. Data are shown as mean ± s.e.m, n=3 (biologically independent experiments).

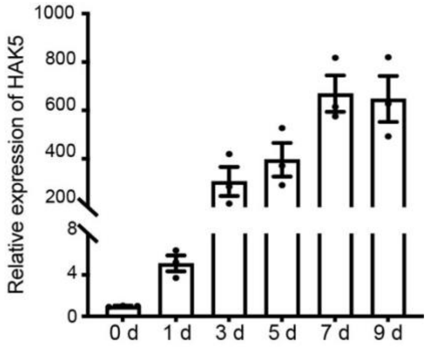

Supplementary Figure 3. Quantitative RT-PCR analysis of HAK5 transcripts upon high- to low-K^+ transfer. Col seedlings were first grown under high-K^+ (20 mM) condition for 4 days and then transferred to low-K^+ (10 μM) medium for the indicated time (d, days). The relative expression of HAK5 was double normalized against the expression level of *ACTIN2* and the expression level from plants grown before low-K^+ transfer. Data are shown as mean ± s.e.m, n=3 (biologically independent experiments).

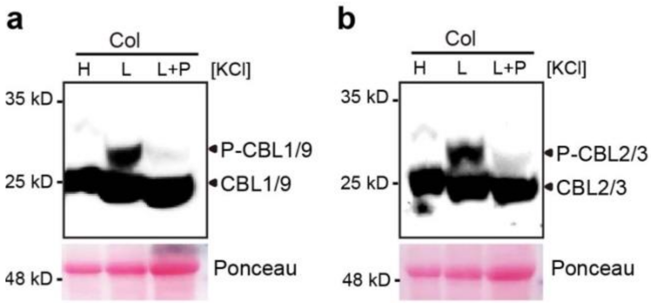

Supplementary Figure 4. Effect of phosphatase treatment on low-K^+-induced mobility shift of CBLs. Total protein was extracted from Col seedlings grown under high-K^+ (20 mM) or low-K^+ (10 μM) for 7 days. The low-K^+ protein samples were treated without or with phosphatase for 30 min before phostag-PAGE analyses, followed by immunoblot with CBL1 antibody (**a**) or CBL3 antibody (**b**). Ponceau staining of the rubisco protein band is shown as a loading control. Each immunoblot result is the representative of at least three repeats.

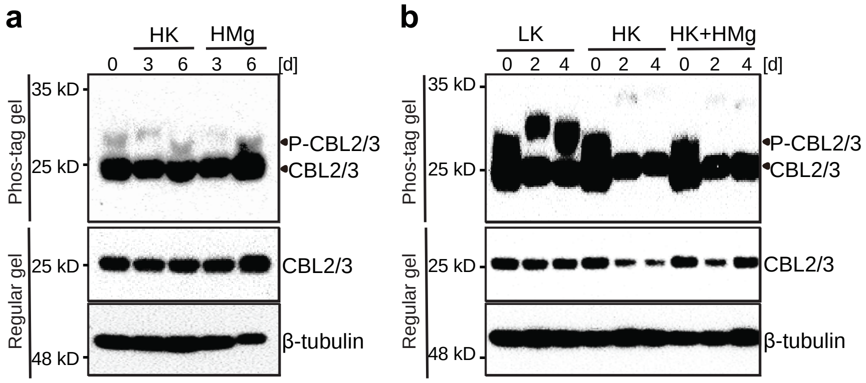

Supplementary Figure 5. High-Mg^{2+} stress fails to induce CBL2/3 phosphorylation. (**a**) CBL2/3 phosphorylation status in Col seedlings after high-K^+ to high-Mg^{2+} transfer. Col seedlings were grown under high-K^+ (20 mM) for 4 days, and then transferred to high-K^+ or high-Mg^{2+} (30 mM $MgCl_2$) for the indicated number of days. (**b**) Changes in CBL2/3 phosphorylation status in Col seedlings upon transfer from low-K^+ to high-K^+ plus high-Mg^{2+}. Col seedlings were grown under low-K^+ (10 μM) for 7 days, and then transferred to high-K^+ (20 mM KCl) or high-K^+ plus high-Mg^{2+} (20 mM KCl plus 30 mM $MgCl_2$) for the indicated number of days. In **a** and **b**, total protein samples were subjected to regular PAGE and phostag-PAGE analyses, followed by immunoblot with CBL3 antibody, and the amount of β-tubulin was determined in parallel as a loading control. Each immunoblot result is the representative of at least three repeats.

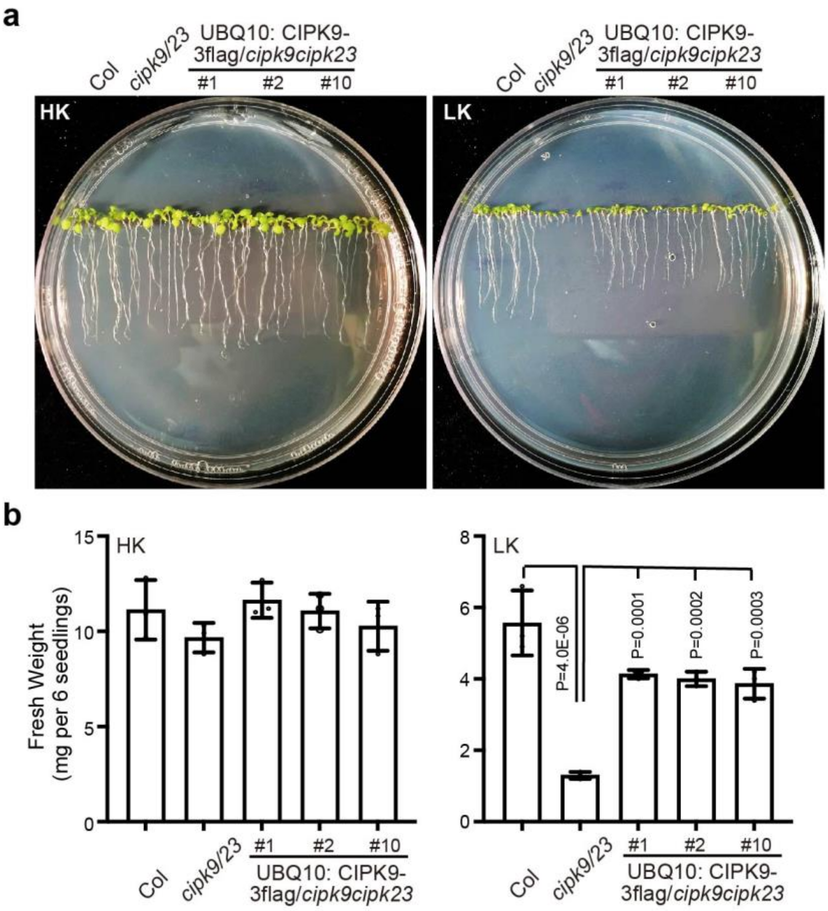

Supplementary Figure 6. UBQ10: CIPK9-3flag complements *cipk9cipk23* phenotypes under low-K^+ stress. (**a**) Representative images of Col, *cipk9/23* double mutant, UBQ10: CIPK9-3flag/*cipk9cipk23* transgenic plants under high- or low-K^+ condition. The seedlings were germinated and grown on the medium containing high-K^+ (20 mM) condition or low-K^+ (10 μM) condition for 7 days. (**b**) Measurement of fresh weight per 6 seedlings at the end of the assay as shown in **a**. Three independent lines were used for each transgenic plant. Data are shown as mean ± SD, n=3 (biologically independent experiments). Statistical analysis between groups were performed by one-way analysis of variance (ANOVA) followed by a Tukey's multiple comparison test.

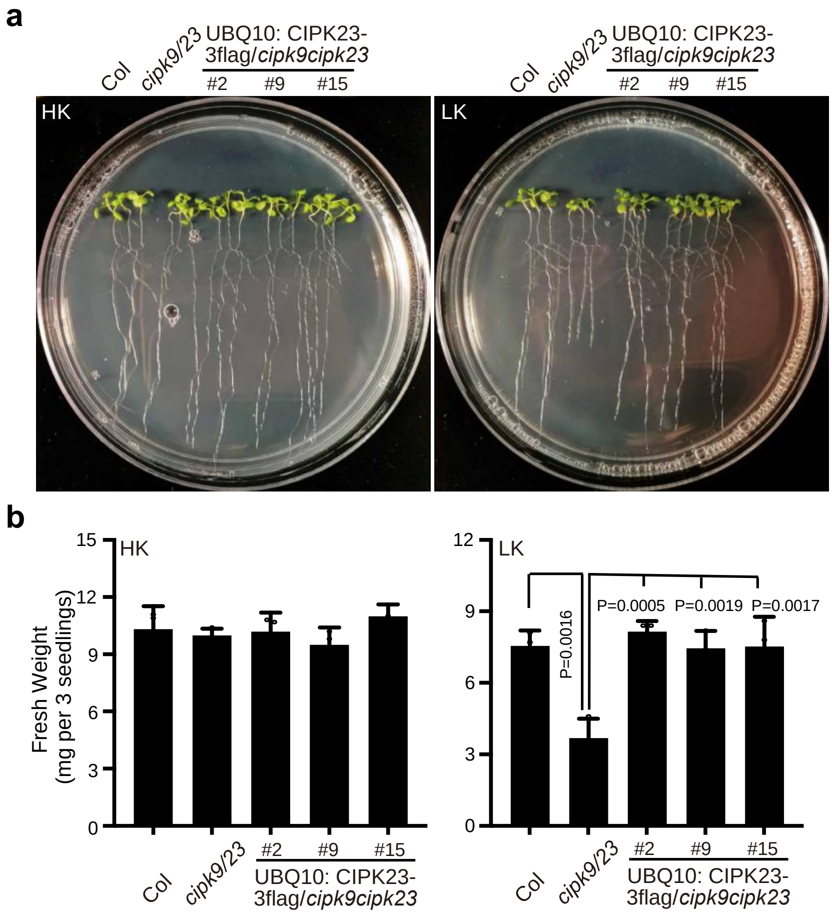

Supplementary Figure 7. UBQ10: CIPK23-3flag complements *cipk9cipk23* phenotypes under low-K^+ stress. (**a**) Representative images of Col, *cipk9/23* double mutant, and UBQ10: CIPK23-3flag/*cipk9cipk23* transgenic plants under high- or low-K^+ condition. The seedlings were grown under sufficient-K^+ (20 mM) condition for 4 days, followed by a transfer to high-K^+ (20 mM) or low-K^+ (10 µM) condition and grown for another 7 days. (**b**) Measurement of fresh weight per 3 seedlings at the end of the assay as shown in **a**. Three independent lines were used for each transgenic plant. Data are shown as mean ± SD, n=3 (biologically independent experiments). Statistical analysis between groups were performed by one-way analysis of variance (ANOVA) followed by a Tukey's multiple comparison test.

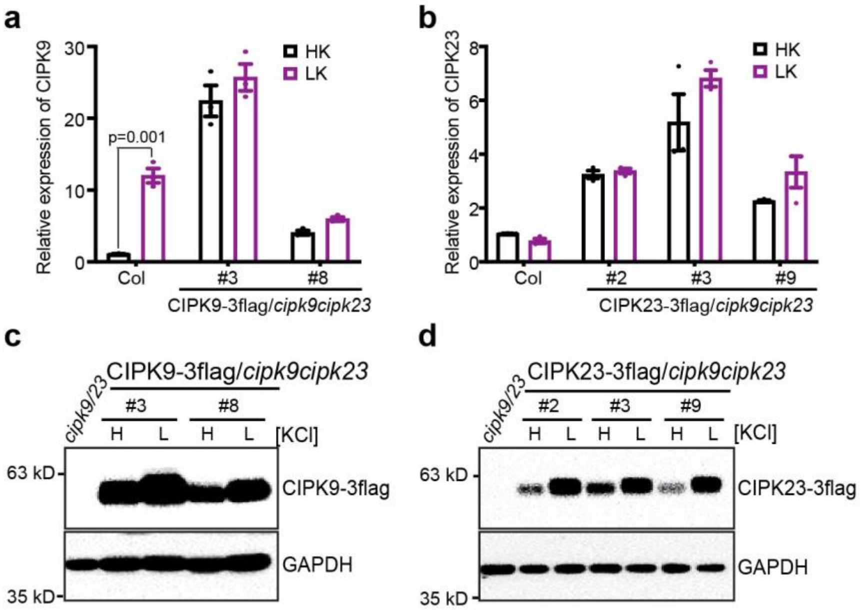

Supplementary Figure 8. (a, b) Quantitative RT-PCR analysis of CIPK9 (**a**) and CIPK23 (**b**) transcripts under high- or low-K^+ condition. The relative expression of each gene was double normalized against the expression level of ACTIN2 and the expression level in plants grown under high-K^+ condition. Data are shown as ± s.e.m, n=3 (biologically independent experiments). (**c, d**) The protein level of CIPK9 and CIPK23 are up-regulated by low-K^+ stress. Two independent transgenic lines of UBQ10: CIPK9-3flag/*cipk9cipk23* (**a, c**) and three independent transgenic lines UBQ10: CIPK23-3flag/*cipk9cipk23* (**b, d**) seedlings were grown under high-K^+ (20 mM) or low-K^+ (10 μM) for 7 days. CIPK9 and CIPK23 proteins are analyzed by immunoblots with flag antibody, and the amount of GAPDH was determined in parallel as a loading control. Each immunoblot result is the representative of at least three repeats.

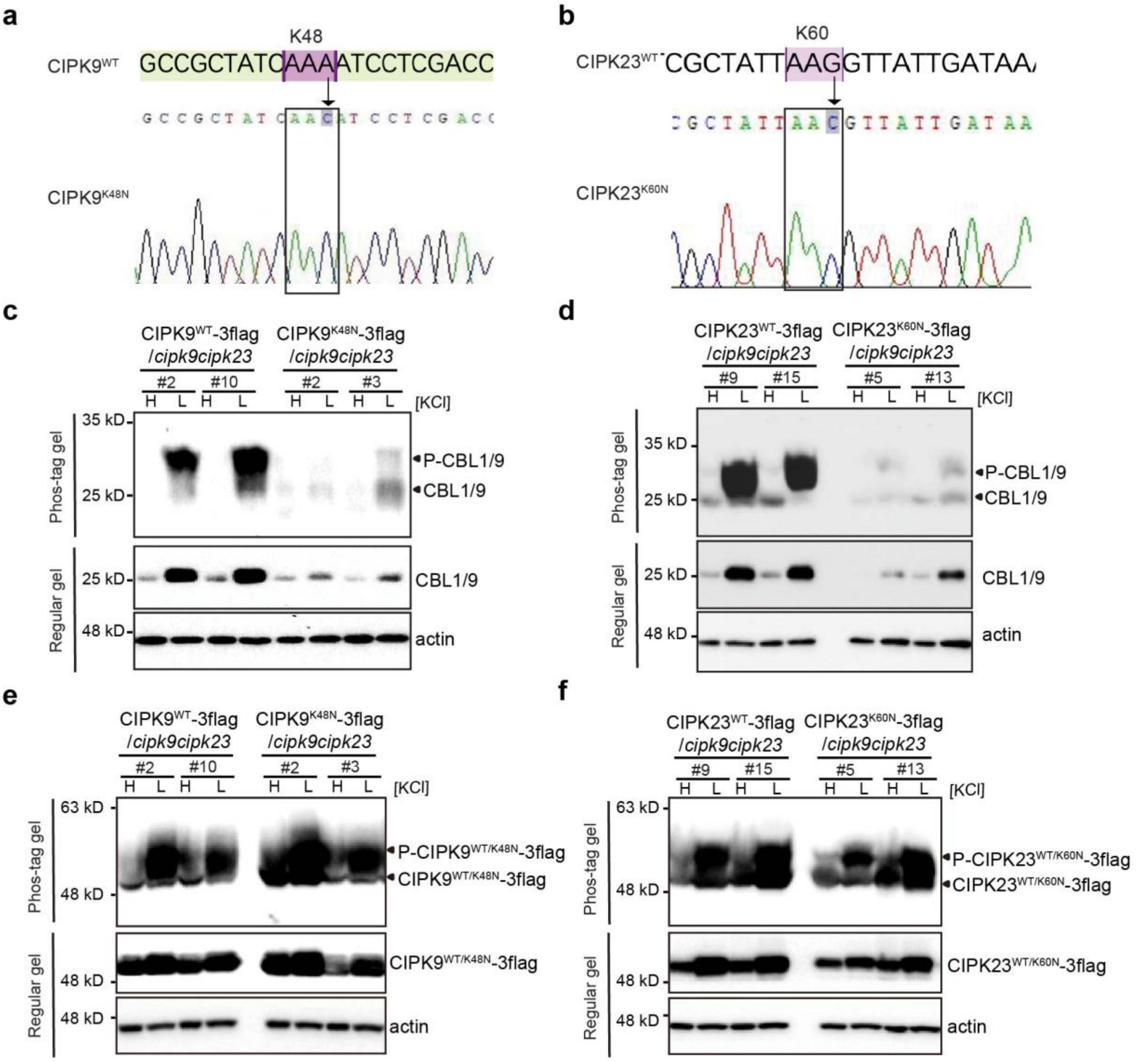

Supplementary Figure 9. The kinase activity of CIPK9 and CIPK23 was ablated in the UBQ10:CIPK9^{K48N}-3flag and UBQ10:CIPK23^{K60N}-3flag plants. (**a**,**b**) Sequencing chromatographs of CIPK9 or CIPK23 PCR products from UBQ10:CIPK9^{K48N}-3flag or UBQ10: CIPK23^{K60N}-3flag plants, demonstrating that the intended K48N or K60N mutation (arrow) was incorporated in the transgenic plants. (**c**) CBL1/9 phosphorylation status and protein level in UBQ10: CIPK9^{WT} and UBQ10: CIPK9^{K48N} seedlings grown under high-K$^+$ (20 mM) or low-K$^+$ (10 μM) for 7 days. (**d**) CBL1/9 phosphorylation status and protein level in UBQ10: CIPK23^{WT} and UBQ10: CIPK23^{K60N} seedlings grown under high-K$^+$ (20 mM) or low-K$^+$ (10 μM) for 7 days. In **c** and **d**, total protein samples were subjected to regular PAGE and phostag-PAGE analyses, followed by immunoblot with CBL1 antibody. The amount of actin was determined in parallel as a loading control. (**e**) Phosphorylation of CIPK9^{WT}-3flag and CIPK9^{K48N}-3flag under high-K$^+$ or low-K$^+$ conditions. (**f**) Phosphorylation of CIPK23^{WT}-3flag and CIPK23^{K60N}-3flag under high-K$^+$ or low-K$^+$ conditions. Proteins of CIPK9-3flag, CIPK23-3flag, CIPK9^{K48N}-3flag and CIPK23^{K60N}-3flag were analyzed using flag antibody. Actin was used as a loading control. From **c** and **f**, two independent transgenic lines were used in each case. Each immunoblot result is the representative of at least three repeats.

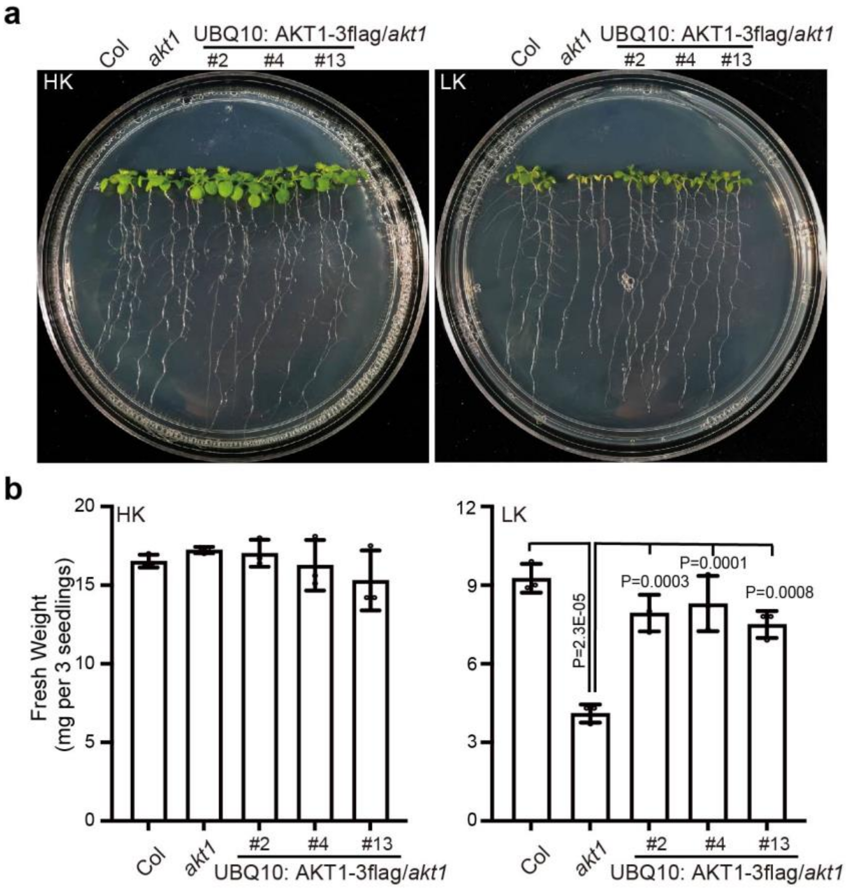

Supplementary Figure 10. UBQ10: AKT1-3flag complements *akt1* mutant phenotypes under low-K^+ stress. (**a**) Representative images of Col, *akt1* mutant, and UBQ10: AKT1-3flag/*akt1* transgenic plants under high- or low-K^+ condition. Seedlings were grown under sufficient-K^+ (20 mM) condition for 4 days, and then were transferred to high-K^+ (20 mM) or low-K^+ (10 μM) condition for another 10 days. (**b**) Measurement of fresh weight per 3 seedlings at the end of the assay as shown in **a**. Three independent lines were used for each transgenic plant. Data are shown as mean ± SD, n=3 (biologically independent experiments). Statistical analysis between groups were performed by one-way analysis of variance (ANOVA) followed by a Tukey's multiple comparison test.

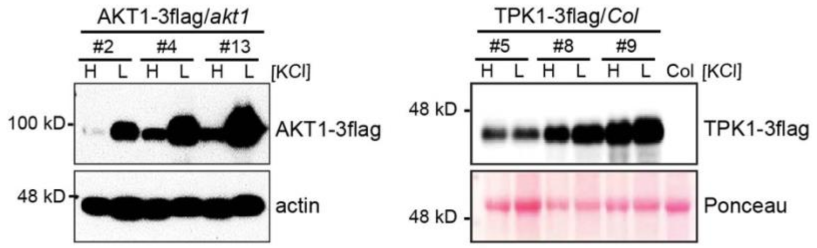

Supplementary Figure 11. AKT1-3flag and TPK-3flag protein amount under high- and low-K^+ conditions. Transgenic plants expressing flag-tagged AKT1 (**a**) or TPK1 (**b**) were grown under high-K^+ (20 mM) or low-K^+ (10 μM) medium for 7 days. Proteins of AKT1-3flag and TPK1-3flag

were analyzed using flag antibody. Actin or ponceau was used as a loading control. Each immunoblot result is the representative of at least three repeats.

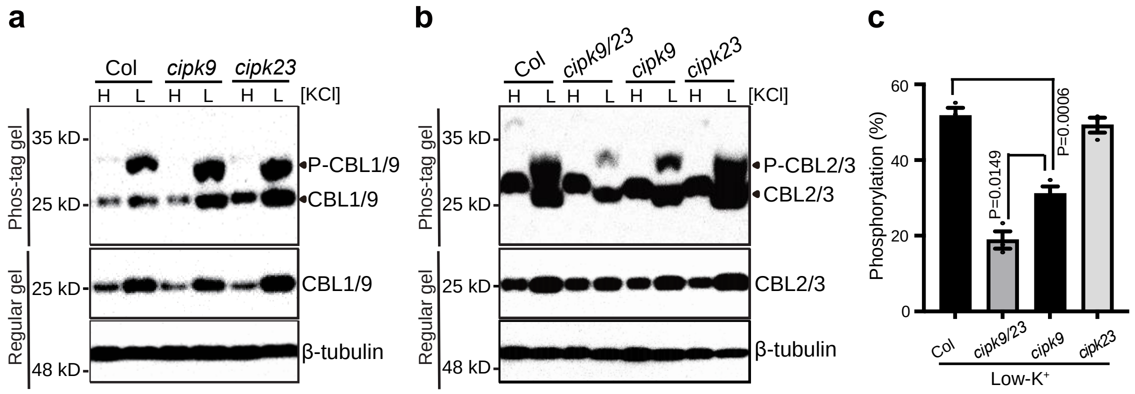

Supplementary Figure 12. CIPK9 and CIPK23 function redundantly in phosphorylating CBL1/9 and CBL2/3 under low-K^+ stress. (**a**) CBL1/9 phosphorylation status in *cipk9* and *cipk23* mutants under high-K^+ or low-K^+ condition. (**b**) CBL2/3 phosphorylation status in *cipk9/23, cipk9* and *cipk23* mutants under high-K^+ or low-K^+ condition. In **a** and **b**, Col, *cipk9/23, cipk9,* and *cipk23* seedlings were grown under high-K^+ (20 mM) or low-K^+ (10 μM) for 7 days and total protein samples were subjected to regular PAGE and phostag-PAGE analyses, followed by immunoblot with CBL1 antibody (**a**) or CBL3 antibody (**b**). The amount of β-tubulin was determined in parallel as a loading control. Each immunoblot result is the representative of at least three repeats. (**c**) Quantification of the CBL2/3 phosphorylation level in Col, *cipk9/23, cipk9,* and *cipk23* seedlings under low-K^+ condition as in **b**. Phosphorylated CBL2/3 level was normalized against total CBL2/3 protein levels. Data are shown as means ± s.e.m., n=3 (biologically independent experiments). P value represents statistically significant differences between groups by one-way analysis of variance (ANOVA) followed by a Tukey's multiple comparison test.

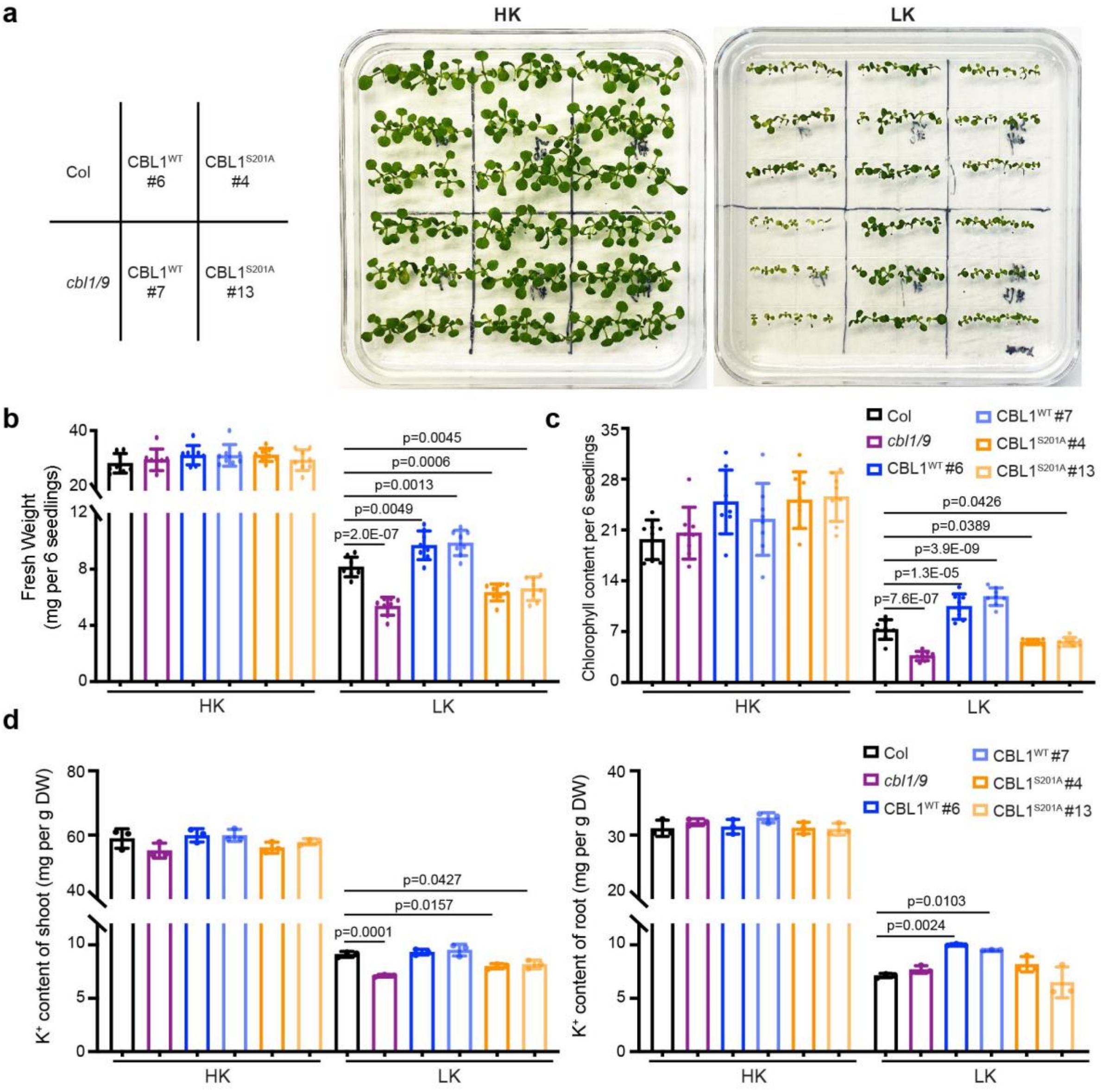

Supplementary Figure 13. CBL1^{S201} phosphorylation is required for CBL1 function. (**a**) Representative images of Col, *cbl1/9* double mutant, and transgenic plants expressing CBL1^{WT}-3flag or CBL1^{S201A}-3flag in the *cbl1cbl9* background. Four-day-old seedlings grown on MS medium were transferred to high-K^+ or low-K^+ medium containing 20 mM NH_4^+ for another 10 days. (**b-d**) Measurement of fresh weight (**b**), chlorophyll content (**c**) per 6 seedlings, and K content (**d**) in the shoot and root tissue at the end of the assay as shown in **a**. Two independent lines were used for each transgenic plant. Data are shown as mean ± SD, n=3 (biologically independent experiments). Statistical analysis between groups were performed by one-way analysis of variance (ANOVA) followed by a Tukey's multiple comparison test.

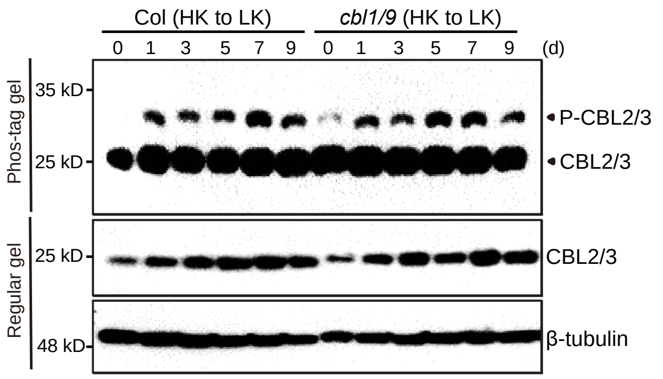

Supplementary Figure 14. CBL1/9 protein level and phosphorylation status in Col and *cbl2/3* double mutant after high- to low-K^+ transfer. Col and *cbl2/3* seedlings were first grown under high-K^+ (20 mM) condition for 4 days and then transferred to low-K^+ (10 μM) medium for the indicated number of days. Total protein samples were subjected to regular-PAGE and phostag-PAGE analyses, followed by immunoblot with CBL3 antibody. β-tubulin was used as a loading control. Each immunoblot result is the representative of at least three repeats.

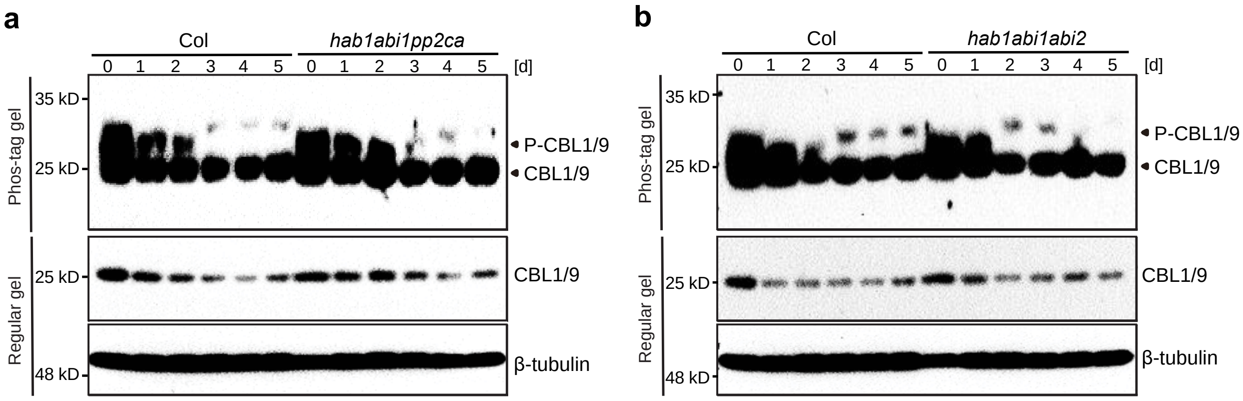

Supplementary Figure 15. CBL1/9 phosphorylation status in *hab1abi1pp2ca* and *hab1abi1abi2* triple mutants after low- to high-K^+ transfer. (**a**) CBL1/9 protein level and phosphorylation status in Col and *hab1abi1pp2ca* triple mutant. (**b**) CBL1/9 protein level and phosphorylation status in Col and *hab1abi1abi2* triple mutant. Col and *hab1abi1pp2ca* or *hab1abi1abi2* triple mutant seedlings were first grown under low-K^+ (10 μM) condition for 7 days and then transferred to high-K^+ (20 mM) medium for the indicated time (d, days). Total protein samples were subjected to regular PAGE and phostag-PAGE analyses, followed by immunoblot with CBL1 antibody. The amount of β-tubulin was determined in parallel as a loading control. Each immunoblot result is the representative of at least three repeats.

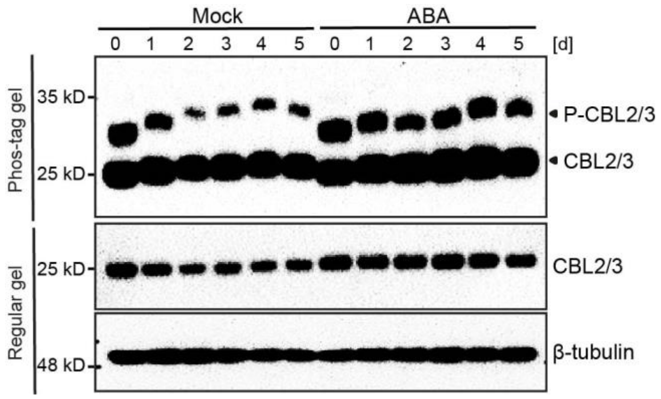

Supplementary Figure 16. The addition of ABA inhibits CBL2/3 dephosphorylation and degradation after low- to high-K^+ transfer. Col seedlings were grown under low-K^+ (10 μM) for 5 days and transferred to high-K^+ (20 mM) in the presence or absence of 5 μM ABA for the indicated number of days. Total protein samples were subjected to regular PAGE and phostag-PAGE analyses, followed by immunoblot with CBL3 antibody. The amount of β-tubulin was determined in parallel as a loading control. Each immunoblot result is the representative of at least three repeats.

Supplementary Table 1. Primers used in this study.

Primers for vector construction	
Primer Name	Sequence (5’ to 3’)
SmaI-CBL1-F	TCCCCCGGGATGGGCTGCTTCCACTCAAA
SpeI-CBL1-R	GGACTAGTTGTGGCAATCTCATCGACCTC
SmaI-CIPK23-F	TCCCCCGGGATGGCTTCTCGAACAACGCC
SpeI-CIPK23-R	GGACTAGTTGTCGACTGTTTTGCAATTGTCC
BamHI-CIPK9-F	CGGGATCCATGAGTGGAAGCAGAAGGAAGGCG
SpeI-CIPK9-R	GGACTAGTTTGCTTTTGTTCTTCAGCGGCTGC
AKT1-F	gagtttttctgattaacagggtaccGCCACCATGAGAGGAG
AKT1-R	cgtggtccttatagtcGGTACCAGAATCAGTTGCAAAGATGAG
CBL1-S201A -F	GTTTCCGGCTTTTGTCTTCCATTCGGAGG
CBL1-S201A -R	CAAAAGCCGGAAACGTTGTCGTAATATCC
SalI-PP2CA-F	ACGCGTCGAC ATGGCTGGGATTTGTTGCG
HindIII-PP2CA-R	CCCAAGCTTTTAAGACGACGCTTGATTATTCCTC
EcoR1-HAB1-F	CCGGAATTCATGGAGGAGATGACTCCCGC
SalI-HAB1-R	ACGCGTCGACTCAGGTTCTGGTCTTGAACTTTCTT
BamH1-ABI1-F	CGCGGATCCATGGAGGAAGTATCTCCGGCG
Sal1-ABI1-R	ACGCGTCGACTCAGTTCAAGGGTTTGCTCTTGAG
EcoR1-ABI2-F	CCGGAATTCATGGACGAAGTTTCTCCTGCA
SalI-ABI2-R	ACGCGTCGACTCAATTCAAGGATTTGCTCTTGA
Primers for quantitative RT-PCR (qRT-PCR)	
Primer Name	Sequence (5’ to 3’)
qCBL1-F	CGA CAT GGA CTG CAC GGG TTA C
qCBL1-R	TCG TGG CAA TCT ACT CGG TCT TA
qCBL9-F	AGC GCC AAG AGG TGA AGC AG
qCBL9-R	TCT CTT TCA CGT CGC AAT CTC G
qHAK5-F	GAGGAGGGGAGGTGGACGAGAC
qHAK5-R	TCTCCTTCCCGACAATTCTTCTTC
qActin2-F	GGAAGGATCTGTACGGTAAC
qActin2-R	GGACCTGCCTCATCATAC
qCBL2-F	GCTCGTGCTCTCTCCGTCTTTC
qCBL2-R	GCCGCTGCTTGCTTTTGCTTTTG
qCBL3-F	CTGAGTCCGGCATGAACCTGTC
qCBL3-R	TTCCCAAATTGTCTCCTCTGCTAA
qCIPK9-F	ATGGCGAGCAAA ACGAAGATC
qCIPK9-R	GCTTTTGTTCTTCAGCGGCTG
qCIPK23-F	ATGGGAGATTGAAGGAAGATGAG
qCIPK23-R	ATCGGTTCCTTCCTCTTTCTCTT
