# Supplementary material for: Potassium nutrient status drives posttranslational regulation of a low-K response network in Arabidopsis
Source: Nat Commun. 2023 Jan 23;14:360. doi: 10.1038/s41467-023-35906-5 (PMC9870859; doi:10.1038/s41467-023-35906-5)
Supplement: Supplementary file 2 — Reporting Summary [file 41467_2023_35906_MOESM2_ESM.pdf]

nature portfolio

Corresponding author(s): Sheng Luan

Last updated by author(s): Dec 21, 2022

Reporting Summary

Nature Portfolio wishes to improve the reproducibility of the work that we publish. This form provides structure for consistency and transparency in reporting. For further information on Nature Portfolio policies, see our Editorial Policies and the Editorial Policy Checklist.

Statistics

For all statistical analyses, confirm that the following items are present in the figure legend, table legend, main text, or Methods section.

n/a	Confirmed	
☐	☒	The exact sample size (n) for each experimental group/condition, given as a discrete number and unit of measurement
☐	☒	A statement on whether measurements were taken from distinct samples or whether the same sample was measured repeatedly
☐	☒	The statistical test(s) used AND whether they are one- or two-sided<br>*Only common tests should be described solely by name; describe more complex techniques in the Methods section.*
☒	☐	A description of all covariates tested
☐	☒	A description of any assumptions or corrections, such as tests of normality and adjustment for multiple comparisons
☐	☒	A full description of the statistical parameters including central tendency (e.g. means) or other basic estimates (e.g. regression coefficient) AND variation (e.g. standard deviation) or associated estimates of uncertainty (e.g. confidence intervals)
☐	☒	For null hypothesis testing, the test statistic (e.g. F, t, r) with confidence intervals, effect sizes, degrees of freedom and P value noted<br>*Give P values as exact values whenever suitable.*
☒	☐	For Bayesian analysis, information on the choice of priors and Markov chain Monte Carlo settings
☒	☐	For hierarchical and complex designs, identification of the appropriate level for tests and full reporting of outcomes
☒	☐	Estimates of effect sizes (e.g. Cohen's d, Pearson's r), indicating how they were calculated

Our web collection on statistics for biologists contains articles on many of the points above.

Software and code

Policy information about availability of computer code

Data collection	WINLAB32 was used to collect ICP-OES data; Opticon Monitor software was used to collect real-time qPCR data;Image Lab was used to collect immunoblots data.
Data analysis	Quantification results of immunoblots were analyzed by Image J software; Microsoft Excel in office 365 and GraphPad Prism 8.0 were used for calculation and statistical analysis of the data; Adobe Illustrator 2021 was used for image assembly.

For manuscripts utilizing custom algorithms or software that are central to the research but not yet described in published literature, software must be made available to editors and reviewers. We strongly encourage code deposition in a community repository (e.g. GitHub). See the Nature Portfolio guidelines for submitting code & software for further information.

Data

Policy information about availability of data

All manuscripts must include a data availability statement. This statement should provide the following information, where applicable:

- Accession codes, unique identifiers, or web links for publicly available datasets
- A description of any restrictions on data availability
- For clinical datasets or third party data, please ensure that the statement adheres to our policy

Provide your data availability statement here.

Human research participants

Policy information about studies involving human research participants and Sex and Gender in Research.

Reporting on sex and gender	*Use the terms sex (biological attribute) and gender (shaped by social and cultural circumstances) carefully in order to avoid confusing both terms. Indicate if findings apply to only one sex or gender; describe whether sex and gender were considered in study design whether sex and/or gender was determined based on self-reporting or assigned and methods used. Provide in the source data disaggregated sex and gender data where this information has been collected, and consent has been obtained for sharing of individual-level data; provide overall numbers in this Reporting Summary. Please state if this information has not been collected. Report sex- and gender-based analyses where performed, justify reasons for lack of sex- and gender-based analysis.*
Population characteristics	*Describe the covariate-relevant population characteristics of the human research participants (e.g. age, genotypic information, past and current diagnosis and treatment categories). If you filled out the behavioural & social sciences study design questions and have nothing to add here, write "See above."*
Recruitment	*Describe how participants were recruited. Outline any potential self-selection bias or other biases that may be present and how these are likely to impact results.*
Ethics oversight	*Identify the organization(s) that approved the study protocol.*

Note that full information on the approval of the study protocol must also be provided in the manuscript.

Field-specific reporting

Please select the one below that is the best fit for your research. If you are not sure, read the appropriate sections before making your selection.

☒ Life sciences ☐ Behavioural & social sciences ☐ Ecological, evolutionary & environmental sciences

For a reference copy of the document with all sections, see nature.com/documents/nr-reporting-summary-flat.pdf

Life sciences study design

All studies must disclose on these points even when the disclosure is negative.

Sample size	No statistical methods were used to predetermine sample sizes. The exact number of samples in each experiment was specified in the figure legends. In all cases, sample sizes were adequate as the results were reproducible between different experimental groups.
Data exclusions	No data were excluded.
Replication	All attempts to replicate the experiments were successful. Each experiment was repeated for at least three times with similar results obtained. Number of repeats in each experiment was provided in the figure legends.
Randomization	Plants of different genotypes were randomly positioned in the growth chamber or in the greenhouse. Randomization was not applied to western blot, ion measurement and qPCR assays.
Blinding	Blinding was impossible because the author who conducted the experiments also performed data acquisition and analysis.

Reporting for specific materials, systems and methods

We require information from authors about some types of materials, experimental systems and methods used in many studies. Here, indicate whether each material, system or method listed is relevant to your study. If you are not sure if a list item applies to your research, read the appropriate section before selecting a response.

Materials & experimental systems

n/a	Involved in the study	
☐	☒	Antibodies
☒	☐	Eukaryotic cell lines
☒	☐	Palaeontology and archaeology
☒	☐	Animals and other organisms
☒	☐	Clinical data
☒	☐	Dual use research of concern

Methods

n/a	Involved in the study	
☒	☐	ChIP-seq
☒	☐	Flow cytometry
☒	☐	MRI-based neuroimaging

Antibodies

Antibodies used	Rabbit polyclonal Anti-CBL1 and Anti-CBL3: custom-produced by Cocalico Biologicals, Inc. (Stevens, PA) ; Anti-β-tubulin: Santa Cruz Biotechnology, SC-166729); Anti-GAPDH: PHYTOAB, PHY0303A; Anti-actin: PHYTOAB, PHY0001; Anti-Flag: Sigma-Aldrich, A8592-2MG); Monoclonal anti-Rabbit secondary antibody: mouse anti-rabbit IgG-HRP provided by Santa Cruz Biotechnology (Cat. No. sc-2357); Monoclonal anti-Mouse secondary antibody: m-IgGκ BP-HRP provided by Santa Cruz Biotechnology (Cat. No. sc-516102).
Validation	Primary antibodies have been validated by the authors' lab using multiple approaches and experiment results with these two antibodies have been published previously. The secondary antibody has been validated by the manufacture.
